# The impacts of different eyes, individual differences, and different time points in healthy rats on the variability of visual electrophysiological examination indicators

**DOI:** 10.3389/fmed.2025.1502787

**Published:** 2025-07-08

**Authors:** Hong Chen, Yu Cheng, Ke Diao, YiFan Wang, Jing An, Haijiang Zhang, RongRong Li, Dan Zhang, SuMian Cheng, Minglian Zhang, LiFei Wang

**Affiliations:** ^1^Hebei Province Key Laboratory of Ophthalmology, Hebei Eye Hospital, Hebei Clinical Research Center for Ocular Diseases, Xingtai, China; ^2^Department of Ophthalmology, Teaching and Research Office, Hebei Medical University, Shijiazhuang, China; ^3^Hebei Xiongxian Hospital, Xiongxian, China

**Keywords:** rat, flash visual evoked potential, full-field electroretinogram, control scheme, animal model

## Abstract

**Purpose:**

This article discussed the repeatability and coefficient of variation (CV) of flash visual evoked potential (FVEP) and full-field electroretinogram (ffERG) indicators of different eyes, individuals, and time points of normal male Sprague-Dawley rats, providing a reference for selecting a reasonable control scheme for retinal and optic nerve disease rat models.

**Methods:**

Twenty normal 6-8 week Sprague-Dawley rats were selected, from which 10 randomly chosen rats underwent ffERG examination and the other 10 underwent FVEP examination. At different time points (1 d, 7 d), Roland visual electrophysiological device was utilized to record in FVEP P2 peak time and N2-P2 amplitude, peak time and amplitude of dark-adapted 0.01 ERG b-wave, peak time and amplitude of dark-adapted 3.0 ERG a- and b-waves, OPs OS2 amplitude, peak time and amplitude of light-adapted 3.0 ERG a- and b-waves, and N1-P1 amplitude of light-adapted 3.0 flash ERG. Meanwhile, we also analyzed the mean ± standard deviation, range, and CV, as well as compared mean ± standard deviation, range, and CV values between two eyes of the same rat and monocular results before and after intervention.

**Results:**

Stable waveforms could be recorded for each rat. Among them, the smallest CV was obtained at the peak time of the FVEP P2 wave (10.1%), while that of amplitude in the VEP P2 wave was relatively large (41.2%). In the ffERG examination, the CV value at each peak time was relatively small (12.9% -39.8%), while the CV value for each wave amplitude was relatively large (33.4% -93.5%). In each waveform, the lower the amplitude, the greater the CV value. By comparing the three control schemes, the CV at the P2 peak time of FVEP examination generated using the baseline ratio approach was the smallest, and that of amplitude calculated using the absolute value approach was the smallest. In the ffERG examination, except for the light-adapted 3.0 ERG a-wave with the lowest CV obtained by the baseline ratio method (57.7%), the CV values of the other examination items were as follows: OD/OS ratio method<absolute value method<baseline ratio method. The CV at each peak time generated by the baseline ratio method was the highest.

**Conclusion:**

In normal adult male Sprague-Dawley rats, the optic nerve function assessment at P2 wave peak time in FVEP is the most stable, and the CV of N2-P2 amplitude is relatively large. During retinal function assessment by ffERG, the detection error for different eyes of the same individual<different individuals<different time points, which can be reduced using a reasonable control scheme.

## 1 Introduction

In ophthalmic animal experiments, assessing visual function is essential for research on various visual disease models and intervention measures for optic nerve and retinal protection. Electrophysiological assessment of visual acuity is currently internationally recognized as the most reliable method for detecting animal vision. However, multiple factors affect the electrophysiological results in animal experiments (1). After unifying significant factors such as anesthetic drugs, animal strains, time, environment, and parameter settings, these parameters still exhibit significant variability. We often find it difficult to determine whether the numerical changes are caused by their own differences or intervention measures, which greatly interfere with the interpretation of experimental results. In previous animal experiments, two distinct measurement methods were often applied for statistical analysis to demonstrate the effectiveness of intervention measures. The first method involves directly statistically analyzing the absolute values of parameters (2, 3), which has the advantage of avoiding errors caused by different time points and the disadvantage of being unable to remove individual differences. The second method is to conduct experiments on the same eye of the same animal, perform a baseline test before the intervention, and statistically analyze the ratio before and after the control experiment (4, 5) which has the advantage of eliminating the influence of different individuals and disadvantage of inability to avoid the influence at various time points. In clinical practice, if the patient’s other eye is healthy, we recommend using the binocular contrast method to eliminate the influence of individuals and time (6). Currently, the impacts of different eyes, individuals, and time points on the visual electrophysiology of rodents are unclear. Therefore, we designed the following experiments to explore the range of variation of electrophysiological parameters in normal SD rats regarding different eyes, individuals, and time points, calculate the coefficient of variation (CV) of three measurement methods, and provide suggestions for selecting the measurement method.

## 2 Materials and methods

### 2.1 Experimental animals

Twenty specific-pathogen-free male Sprague–Dawley rats, aged 6–8 weeks and weighing approximately 200–250 g, were commercially available from Beijing Vital River Laboratory Animal Technology Co., Ltd. (Beijing, China) and housed at the Animal Experiment Center of Hebei Eye Hospital at a constant temperature of 25°C. The reason for selecting male rats is that the electroretinography (ERG) amplitude of female rats gradually increases from 60 to 200 days postnatally, and is influenced by the estrous cycle. In contrast, the ERG amplitude of male rats remains stable. They were given free access to water and maintained on 12 h/12 h light/dark cycles. An anesthetic overdose carries out the euthanasia of animals. The experimental procedures strictly adhered to the Statement of Animal Use issued by the Association for Research in Vision and Ophthalmology regarding the use of animals in ophthalmology and visual research. All animal experiments were conducted under the approval and supervision of the Institutional Animal Care and Use Committee of Hebei Eye Hospital (ethics approval no.: 2024LW01).

### 2.2 ffERG examination

Ten rats were completely acclimated to darkness for 10 h, after which compound Tropicamide Eye drops (Alcon Laboratories, United States) were administered to dilate the pupils before the examination, and 1.2% tribromoethanol (10 mL/kg, 20,240,105) was employed for anesthesia. The body temperature of the rats was maintained using the Roland visual electrophysiological device (Roland Consult Stasche & Finger GmbH, Germany) and the Roland animal-specific test bench and heating plate, both of which were operated by the same experienced physician. Electroretinography was conducted according to the standards established by the International Society for Clinical Electrophysiology of Vision (ISCEV).

#### 2.2.1 Dark-adapted ERG recording protocol

All procedures were performed under red light-emitting diode (LED) illumination in a darkroom between 6:00 and 10:00 p.m. A corneal ring electrode (ERG recording) was placed after topical anesthesia with Alcaine^®^ (proxymetacaine hydrochloride 0.5%, Alcon Laboratories), ensuring stable contact with the corneal surface. Reference and ground electrodes consisted of subdermal needle electrodes positioned near the mouth and tail base, respectively (7), with impedance maintained at <10 kΩ. Full-field stimuli were delivered using a Ganzfeld LED system (Roland Consult Stasche & Finger GmbH, Germany) (background luminance: 0 cd·s/m^2^). Signal filtering included a 0.1 Hz high-pass and 500 Hz low-pass cutoff. White flashes (0.01–3.0 log cd·s/m^2^ intensity; 2–10 s interstimulus interval) were administered to elicit dark-adapted responses.

#### 2.2.2 Indicators of ffERG

After dark adaptation, light adaptation lasted for 10 min. During light acclimatization to 3.0 ERG, the stimulus intensity was 3 log cd·s/m^2^ with a flash interval of 0.5 s. Both eyes were simultaneously tested, and the above-mentioned examinations were repeated after 7 days. Observation indicators: Based on the standards recorded by ISCEV ERG and the expert consensus on Clinical Electrophysiology of Vision related terminology in China (8), 5 commonly used indicators of ffERG were recorded: peak time and amplitude of dark-adapted 0.01 ERG b-waves, peak time and amplitude of 3.0 ERG a- and b-waves, OS2 amplitude of oscillatory potentials (OPs), peak time and amplitude of light-adapted 3.0 ERG a- and b-waves, N1-P1 amplitudes of light-adapted 3.0 flash ERG, amplitude: trough-to-peak amplitude. Peak time: The time from the beginning of a stimulus to the peak or trough, also referred to as implicit time.

### 2.3 FVEP examination

The other 10 rats underwent flash visual evoked potentials (FVEP) examination without dark adaptation or pupil dilation. The needle-shaped electrodes were used as visual evoked potential (VEP) electrodes and placed at the midpoint between the two eyes with forward insertion by 1–1.5 cm. The reference electrode and grounding electrode were set in the same way as the ERG examination. The contralateral eye was covered with an eye mask. A white flash stimulus (5 dB, 9.49 cd·s/m^2^) was analyzed at a frequency of 1.0 Hz. The band-pass filtering was achieved using the recommended band-pass from 0.5 to 50 Hz, 100 times in total. Then, 3 stable waveforms were recorded every 5 min. The remaining operations and preparations were identical to those in Section 1.2. Seven days later, the above examinations were repeated. Observation indicators: P2 peak time and N2–P2 amplitude in F-VEP.

### 2.4 Data analysis

With the use of statistical software SPSS 26.0, the data were analyzed using three measurement methods (absolute value, Oculus Dexter/Oculus Sinister (OD/OS) ratio, baseline ratio) and summarized as mean ± standard deviation, range, and coefficient of variation (CV). Among them, the first right eye examination data were recorded as the absolute value; the first right/left eye examination data were expressed as OD/OS ratio; the first examination data were the baseline data, and the baseline ratio presented the second value/baseline value measured 7 days later.

## 3 Results

### 3.1 Characteristics and dispersion of FVEP and ffERG values in normal male Sprague–Dawley rats

**Figure 1:**
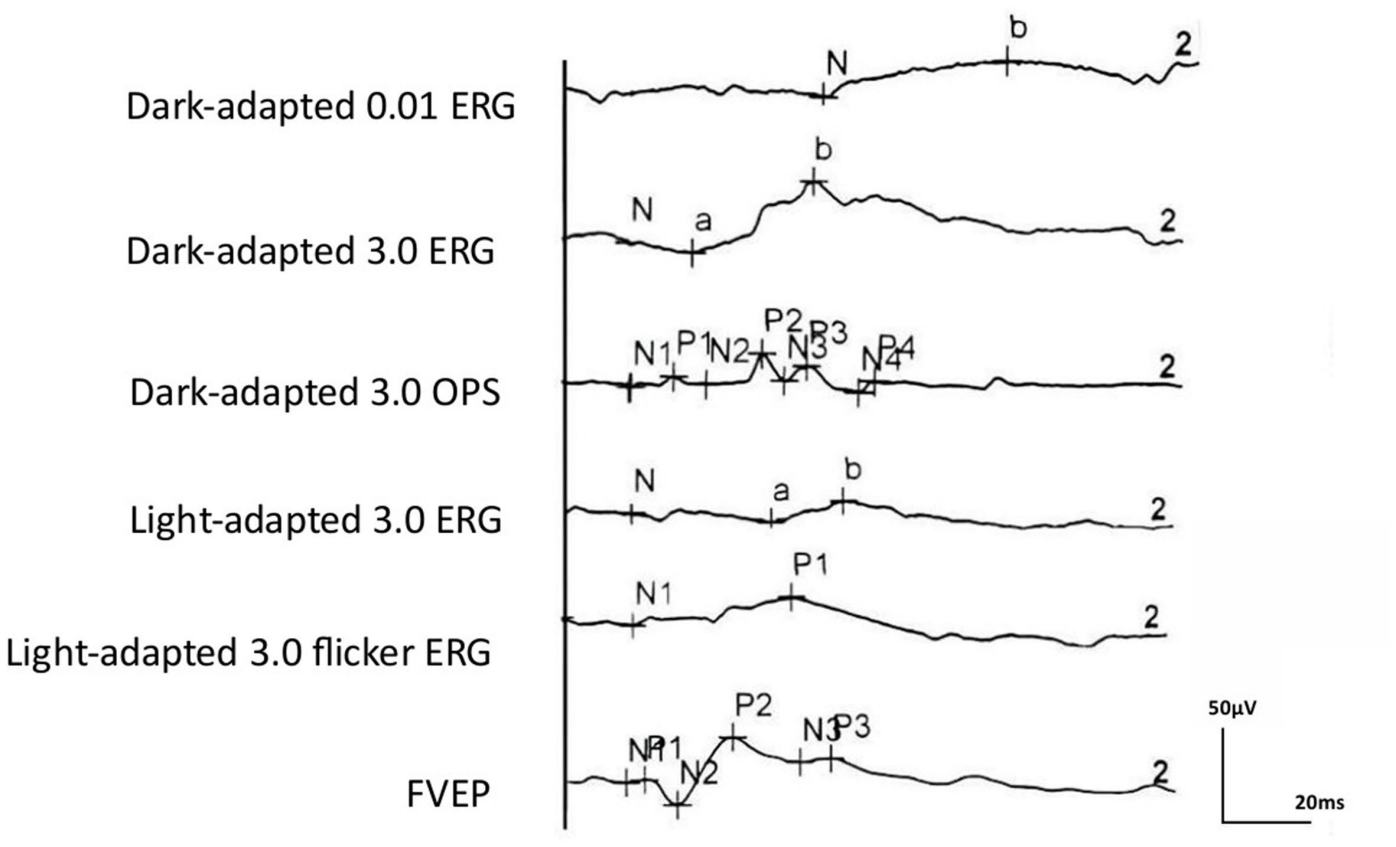
ERG and VEP waveforms of healthy SD rats.

**Figure 2:**
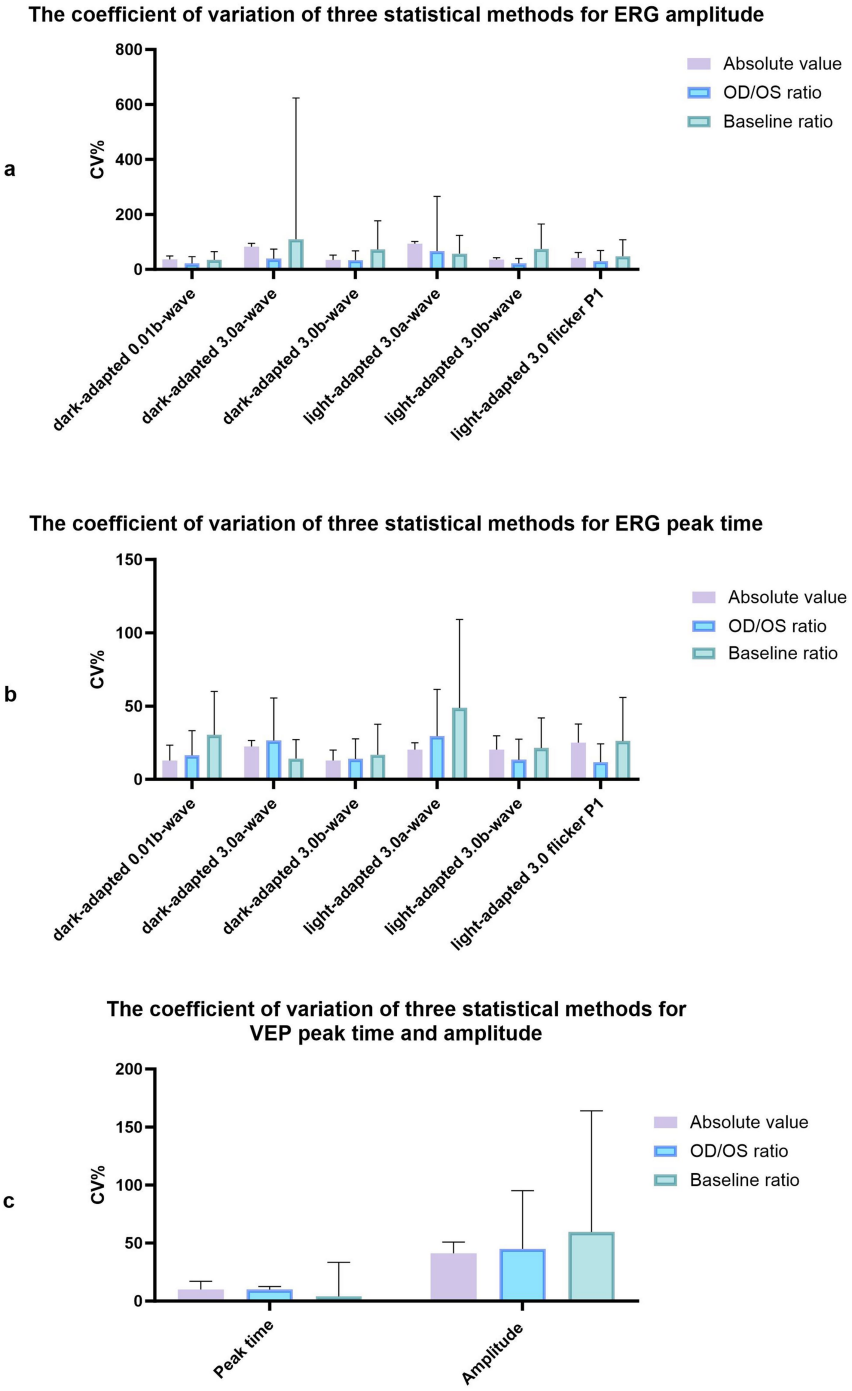
The coefficient of variation of three statistical methods for amplitude and peak time of ERG and VEP. **(a)** The coefficient of variation of three statistical methods for ERG amplitude. **(b)** The coefficient of variation of three statistical methods for ERG peak time. **(c)** The coefficient of variation of three statistical methods for VEP peak time and amplitude.

Regular ERG and VEP waveforms were generated for all rats. Compared with the b-wave of dark-adapted 3.0, the a- and b-waves of light-adapted 3.0 ERG and waveforms of light-adapted 3.0 flash ERG were significantly low and flat, as depicted in Figure 1. The Shapiro–Wilk test method was used to conduct a normal-distribution test on all data—all data followed the normal distribution. In the examination of different items of dark-adapted and light-adapted FVEP and ffERG, significant differences existed in the coefficients of variation among different rats at the same time point. The CV value for the peak time of FVEP P2 waves was the smallest (10.1%), while that for the N2–P2 amplitude was relatively large (41.2%). The CV at each peak time of ffERG was relatively small (12.9–39.8%), while the CV for each amplitude was relatively large (33.4–93.5%). Among them, the CV for the amplitude of the light-adapted 3.0 ERG a-wave was the highest (93.5%), followed by that for the amplitude of the dark-adapted 3.0 ERG a-wave (89.7%). In all waveforms, the lower the amplitude, the greater the CV value. At the same time point, the CV values of the same rat were similar between the two eyes. The dark-adapted and light-adapted 3.0 ERG a-waves had relatively low amplitudes but the highest CV values. See Figures 2a,b for details.

### 3.2 The CV values in normal Sprague–Dawley rats calculated using three measurement approaches (absolute value, right/left eye ratio at the same time point, and ratio of the same eye at different time points)

**Figure 3:**
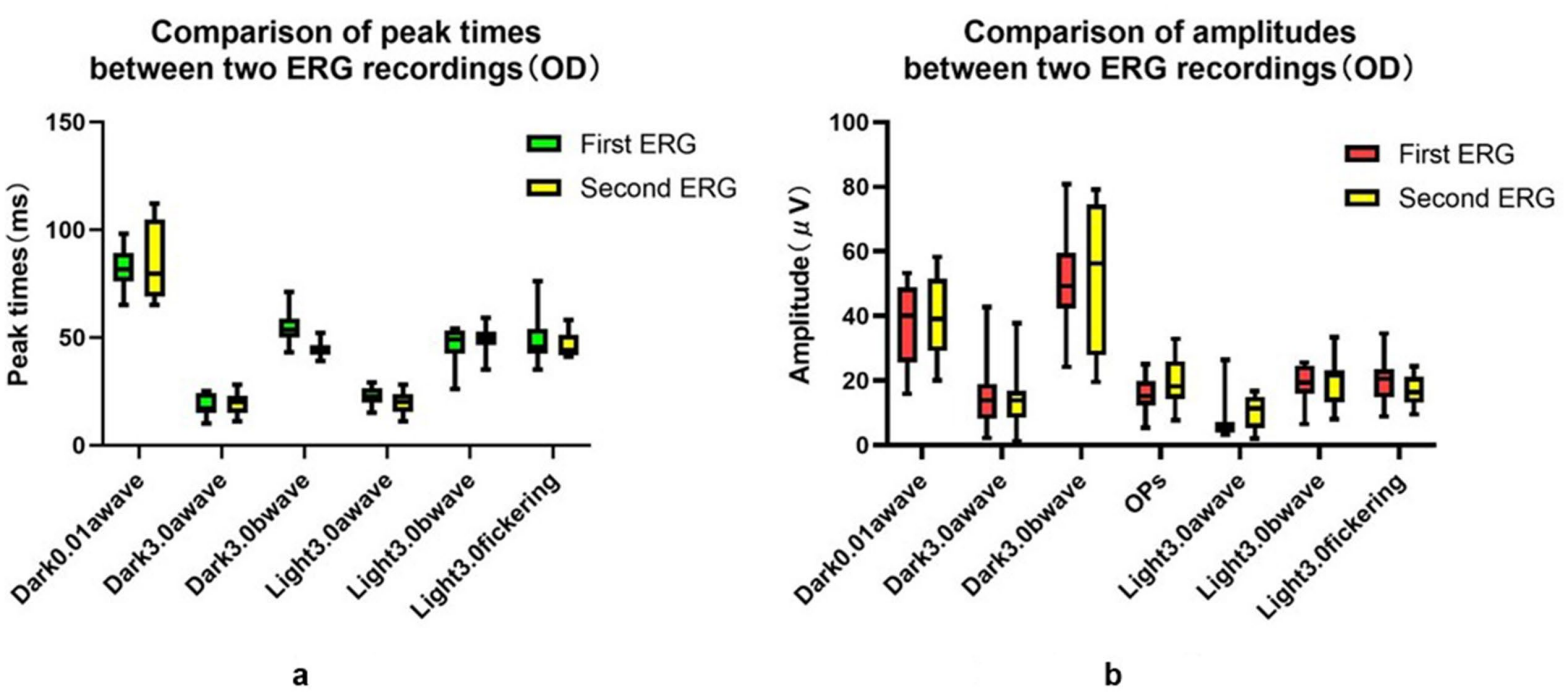
Comparison of peak time and amplitudes between two ERG recordings (OD). **(a)** Comparison of peak time between two ERG recordings (OD). **(b)** Comparison of amplitudes between two ERG recordings (OD).

The examination data varied among different rats, as well as between the left and right eyes of the same rat, and within the same rat at different time points. The CV values obtained using the three measurement approaches were summarized in Figure 2A. The CV value at the peak time of FVEP P2 waves obtained using the baseline ratio approach was the smallest, and that of the amplitude calculated using the absolute value approach was the smallest. In the ffERG examination, except for the light-adapted 3.0 ERG a-wave with the smallest CV obtained by the baseline ratio method (57.7%), the CV values of the other examination items were ranked from small to large as follows: OD/OS ratio method < absolute value method < baseline ratio method. The CV values at each peak time were relatively small, with the absolute value method (12.9–25.1%) and the baseline ratio method yielding the highest CV values (see Figure 3; Table 1).

**Table 1:** Peak time and amplitude of ERG and VEPP2 in healthy rats using different statistical methods.

Statistical methods	Number of eyes	Adaptation 0.01 b-wave						Dark adaptation 3.0 a-wave					
		Peak time (x̅ ± s)	Range	Coefficient of variation%	Amplitude (x̅ ± s)	Range	Coefficient of variation%	Peak time (x̅ ± s)	Range	Coefficient of variation%	Amplitude (x̅ ± s)	Range	Coefficient of variation %
Absolute value	10	81.29 ± 10.48	33.00	12.9	34.97 ± 12.69	31.5	36.3	18.14 ± 4.09	12.00	22.5	15.64 ± 12.73	40.53	82.4
OD/OS ratio	10	101.43 ± 16.72	42.70	16.5	107.86 ± 24.05	68.8	22.3	109.12 ± 28.96	93.09	26.5	90.90 ± 35.31	115.68	38.8
Baseline ratio	10	96.91 ± 29.48	71.78	30.4	85.44 ± 29.91	91.28	35.0	91.32 ± 12.98	36.36	14.2	466.85 ± 513.82	1172.31	110

Statistical methods	Number of eyes	Dark adaptation 3.0 b-wave						Dark adaptation 3.0 b-wave					
		Peak time (x̅ ± s)	Range	Coefficient of variation %	Amplitude (x̅ ± s)	Range	Coefficient of variation %	Peak time (x̅ ± s)	Range	Coefficient of variation %	Amplitude (x̅ ± s)	Range	Coefficient of variation %
Absolute value	10	54.71 ± 7.07	24.00	12.9	52.4 ± 17.99	55.6	34.3	23.29 ± 4.71	14.00	20.2	8.60 ± 8.04	23.16	93.4
OD/OS ratio	10	97.39 ± 13.62	93.11	14.0	100.16 ± 33.76	100.25	33.7	108.20 ± 31.93	96.41	29.5	299.69 ± 199.30	596.52	66.5
Baseline ratio	10	124.28 ± 20.85	60.57	16.8	144.02 ± 104.61	169.75	72.6	123.81 ± 60.33	184.69	48.7	114.32 ± 66.00	146.05	57.7

Statistical methods	Number of eyes	Light adaptation 3.0 b-wave						Light adaptation 3.0 flickering P1					
		Peak time (x̅ ± s)	Range	Coefficient of variation %	Amplitude (x̅ ± s)	Range	Coefficient of variation %	Peak time (x̅ ± s)	Range	Coefficient of variation %	Amplitude (x̅ ± s)	Range	Coefficient of variation %
Absolute value	10	46.29 ± 9.39	28.00	20.3	17.67 ± 6.38	18.88	36.1	50.29 ± 12.60	41	25.1	20.02 ± 19.45	25.70	41.9
OD/OS ratio	10	104.45 ± 14.03	32.40	13.4	87.62 ± 17.90	58.20	21.7	103.05 ± 12.05	29.9	11.7	124.97 ± 38.19	113.40	30.5
Baseline ratio	10	95.99 ± 20.52	67.14	21.4	121.16 ± 90.62	280.60	74.8	113.49 ± 29.68	87.65	26.2	125.18 ± 60.23	172.60	48.1

## 4 Discussion

Visual electrophysiological examination is the most important indicator reflecting visual function in ophthalmic animal experiments. Because it does not require the examinee’s subjective manipulation or drug injection, this examination has the advantages of objectivity, noninvasiveness, and quantification. It often serves as a crucial measurement indicator for constructing animal models of retinal and optic nerve diseases and exploring therapeutic plans (6). With the use of flash stimuli, FVEP extracts electrophysiological signals from the occipital cortex of the brain and evaluates the function of the visual pathway (9). After more than 100 years of research, the origin of each component of ffERG is now relatively clear. ffERG reflects the overall retinal function and stratifies the functions of retinal the rod-like and cone-like cell pathways, as well as the functions of the outer and inner retina (1). Electrophysiological examination is an important measurement indicator in the animal modeling and treatment of diseases including glaucoma (10, 11) retinal ischemic diseases (12–14), neurodegenerative diseases (15), optic nerve injury (2), retinal light damage (16, 17), and high-altitude retinopathy (18).

Because visual electrophysiological values are influenced by various factors, excluding laboratory environment, anesthesia, animal body temperature, dark adaptation time, parameter settings, recording electrodes, among others, significant differences still persist at different time points and among individuals. Each rat requires 10–15 min for a dark adaptation test after baseline stabilization and 10 min for a light adaptation test before various tests. Thus, the number of samples detected by the same operator and the same instrument each time is limited. In statistics, the sample size *n* = Z^2^ × *σ*^2^/d^2^, and CV = σ/*μ*. The larger the CV, the greater the degree of data dispersion, and the larger the required sample size. In animal basic studies, the success of disease models and the effectiveness of intervention measures often require evidence from changes in visual function. Generally, researchers compare the visual electrophysiological measurement results between the experimental (or intervention) group and the control group. Therefore, the selection of the control group is quite important. Individual differences among different rats cannot be avoided if we choose rats without any intervention measures as the controls; Detection differences at different time points are inevitable when we use the same eye of the same rat as controls for comparisons before and after intervention. In clinical practice, we often compare the eyes of the same patient with normal eye waveforms as controls to reduce potential differences among different individuals and at different time points (6). Therefore, we need to clarify the error size caused by three factors: different individuals at the same time point, different eyes of the same individual, and different time points of the same individual, to obtain more reliable data and select the most reasonable control scheme.

Currently, there are few studies on the influence of these three factors on visual electrophysiological values in healthy rats. Zhao et al. (19) investigated the a-, b-, and OPs-waves of FERG in different healthy rats and found significant differences among individuals. Bui et al. (20) believed that rats had smaller a- and b-waves in dark-adapted and light-adapted ERGs than humans, and individual differences existed. However, there is a lack of comparisons at different time points and different eyes. Zhang et al. (21) believed that visual electrophysiological examination, as a functional indicator, exhibited individual differences, and even individual functional states varied at different recording times, which cannot be entirely avoided. Therefore, it is necessary to minimize the animal’s influence on the experiment during study design. The aforementioned studies did not specifically compare individual differences in animals, as well as state differences at different time points and eyes.

In this research, we conducted two electrophysiological tests at different time points with the same group of healthy rats, operated by the same operator, using the same instrument, within the same dark adaptation time, under the same anesthesia and parameter settings. We summarized the data characteristics and dispersion degree and calculated the CV values of the results using three control strategies. For variables following a normal distribution, the statistical indicators that describe their dispersion trend include range (maximum, minimum), mean, standard deviation, and CV (standard deviation/mean * 100%). All data conformed to the normal distribution. In the FVEP examination, the CV at the P2 peak time was extremely small, and all three control methods yielded CV values of less than 10.1%. The CV for the amplitude obtained using the absolute value method was the smallest (41.2%). In the ffERG examination, the CV at peak time was relatively low, and the CV values for the amplitudes of all waves were relatively high; the lower the amplitude, the higher the CV value. The amplitudes of dark-adapted 3.0 ERG a-wave and light-adapted 3.0 ERG a-wave had extremely high CV values (71.4–93.5%). It is not recommended as an indicator for visual function test, considering that the retina of rats is mainly composed of rod-like cells and has a low abundance of cone-like cells (22). With the application of different control methods, except for the light-adapted 3.0 ERG a-wave, the calculated CV values of other examination items were ranked as follows: OD/OS ratio method<absolute value method<baseline ratio method. If experimental conditions permit, the normal eye of the same rat can be considered as the control group, and the OD/OS ratio method can be applied for comparison to reduce experimental errors.

Limitations also exist in this research. Although the OD/OS ratio method yields the smallest CV, with the normal eye from the same rat serving as the control, this method is only applicable for studies on local modeling or treatment of the eyes. Additionally, drugs entering the vitreous cavity of one eye may also have specific effects on the contralateral eye after metabolism, making such studies unsuitable. Given that systemic interventions (e.g., pharmacological metabolism) may induce simultaneous effects on bilateral visual function, the application of the OD/OS ratio analysis is methodologically limited in such experimental paradigms. Due to the complex impact of various pathological conditions on the eyes, we only measured the values of healthy rats. We did not conduct an in-depth exploration of the visual electrophysiological values of rats under pathological conditions.

## Data Availability

The original contributions presented in the study are included in the article/Supplementary material, further inquiries can be directed to the corresponding author.
