# Supplementary material for: The impacts of different eyes, individual differences, and different time points in healthy rats on the variability of visual electrophysiological examination indicators
Source: Front Med (Lausanne). 2025 Jul 8;12:1502787. doi: 10.3389/fmed.2025.1502787 (PMC12279882; doi:10.3389/fmed.2025.1502787)
Supplement: Supplementary file 1 [file Data_Sheet_1.pdf]

RETIport32	HEBEI EYE HOSPITAL<br>(No.399 Quanbeidong Rd.xingtai)		
	Patient: ZC-CEHN-1, CHen, 7/11/2023<br>Tested: 7/12/2023 6:31:12 PM<br>ID:	Sex/Age: M/0<br>Operator:	Electrode: NEEDLE Thread<br>Pupil Size: 2 dil.

Scotopic 0.01 ERG

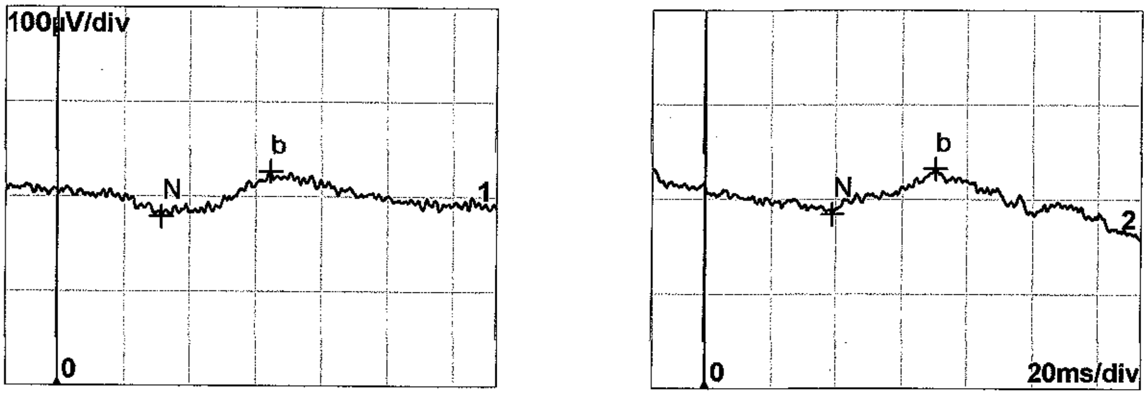

Channel	b [ms]	b-wave
1 R-1	65	47.4µV
2 L-2	71	47.9µV

Scotopic 3.0 ERG

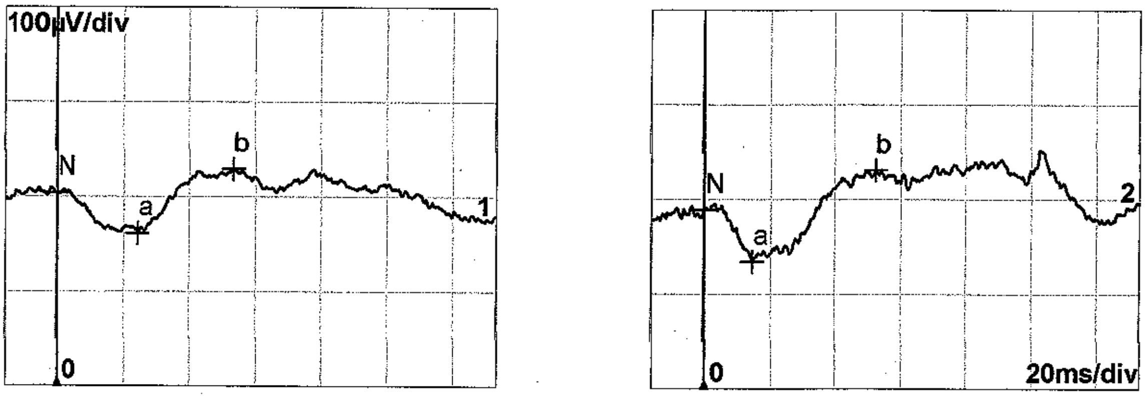

Channel	a [ms]	b [ms]	a-wave	b-wave	b/a
1 R-1	25	54	42.7µV	67.5µV	1.6V
2 L-2	15	53	54µV	96.2µV	1.8V

Scotopic 3.0 oscillatory potentials

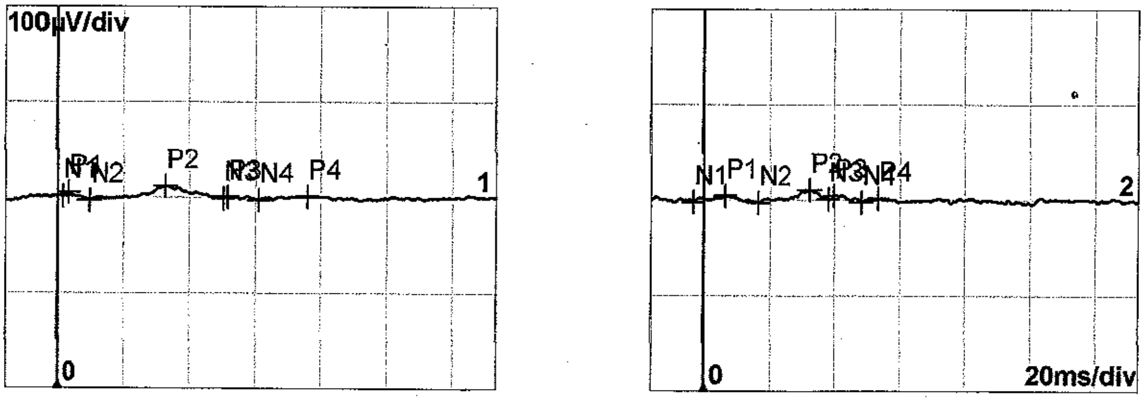

Channel	N1 [ms]	P1 [ms]	N2 [ms]	P2 [ms]	N3 [ms]	P3 [ms]	N4 [ms]	P4 [ms]	OS1	OS2	OS3	OS4	Total
1 R-1	2	4	10	33	51	52	61	76	3.91µV	15.3µV	1.59µV	3.98µV	0.000V
2 L-2	-3	6	17	33	38	40	48	54	6.88µV	13.6µV	3.74µV	3.76µV	0.000V

RETIport32	HEBEI EYE HOSPITAL<br>(No.399 Quanbeidong Rd.xingtai)		
	Patient: ZC-CEHN-1, CHen, 7/11/2023<br>Tested: 7/12/2023 6:31:12 PM<br>ID:	Sex/Age: M/0<br>Operator:	Electrode: NEEDLE Thread<br>Pupil Size: 2 dil.

Photopic 3.0 ERG

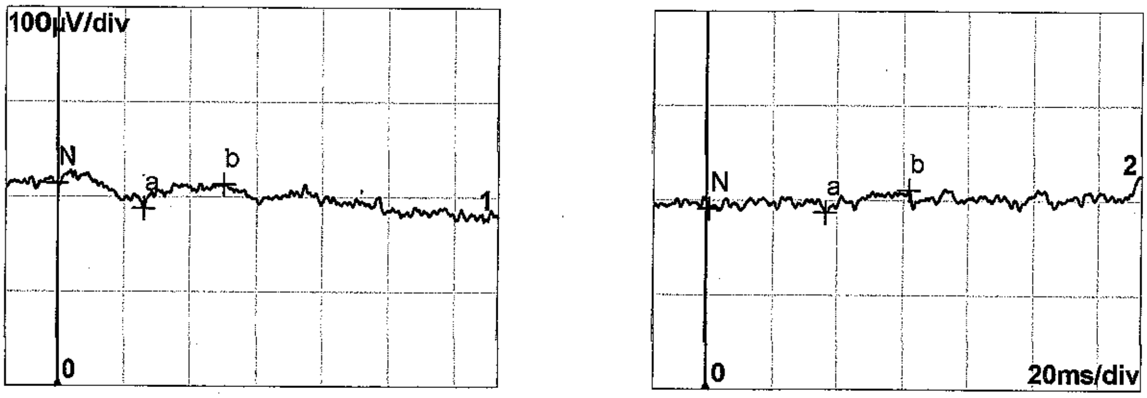

Channel	a [ms]	b [ms]	a-wave	b-wave
1 R-1	26	50	26.4µV	25.3µV
2 L-2	37	62	3.91µV	23.6µV

Photopic 3.0 flicker

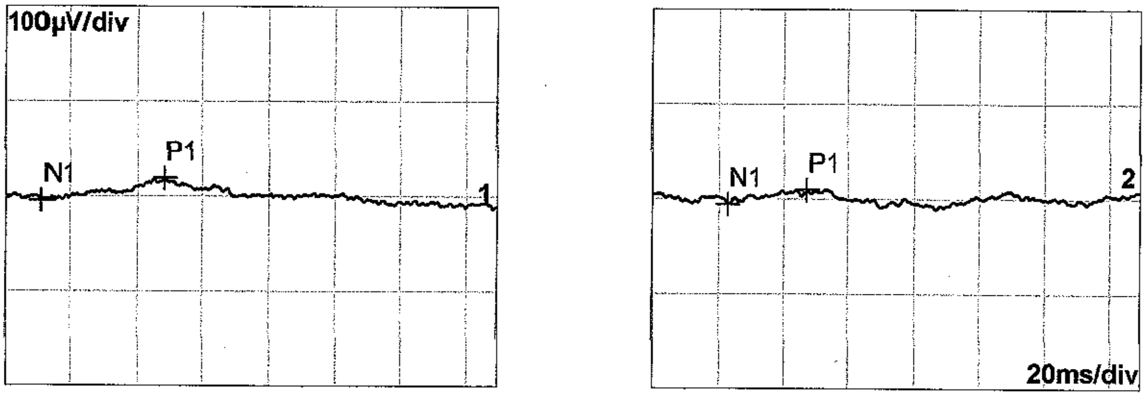

Channel	N1 [ms]	P1 [ms]	V1	N1-P1
1 R-1	12	49	5.04µV	23.4µV
2 L-2	23	48	10.9µV	15.5µV

RETIport32

HEBEI EYE HOSPITAL
(No.399 Quanbeidong Rd.xingtai)

Patient:	ZC-CEHN-3, CHen, 7/11/2023		Electrode: NEEDLE Thread
Tested:	7/12/2023 9:34:17 PM	Sex/Age: M/0	Pupil Size: 2 dil.
ID:		Operator:	

Scotopic 0.01 ERG

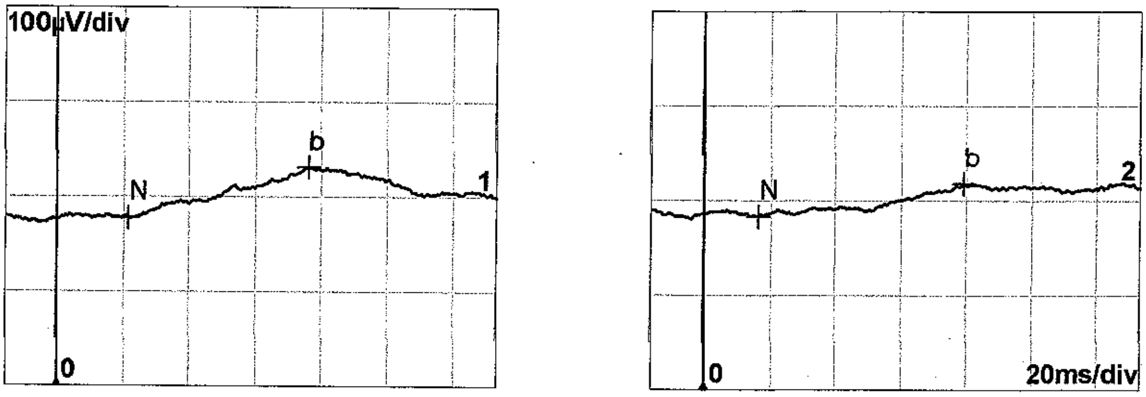

Channel	b [ms]	b-wave
1 R-1	77	53.3µV
2 L-2	80	34.6µV

Scotopic 3.0 ERG

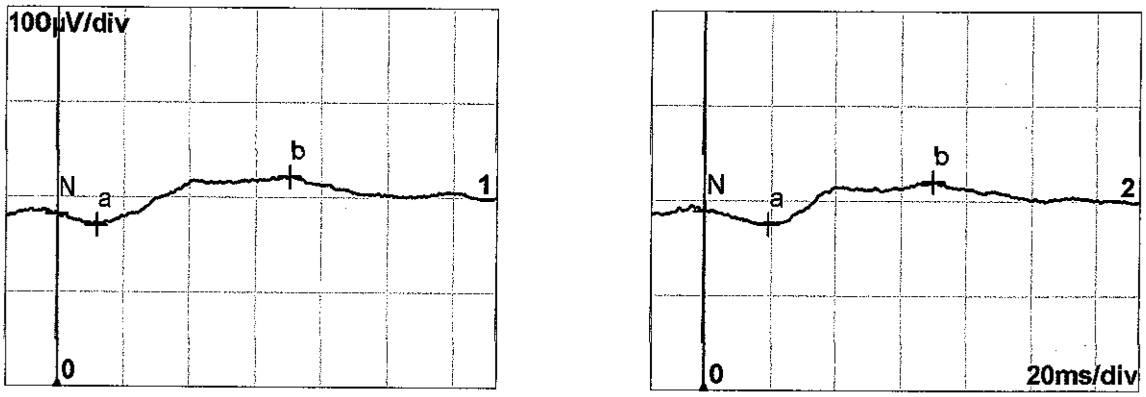

Channel	a [ms]	b [ms]	a-wave	b-wave	b/a
1 R-1	12	71	11.6µV	51.3µV	4.4V
2 L-2	19	70	13.5µV	44.8µV	3.3V

Scotopic 3.0 oscillatory potentials

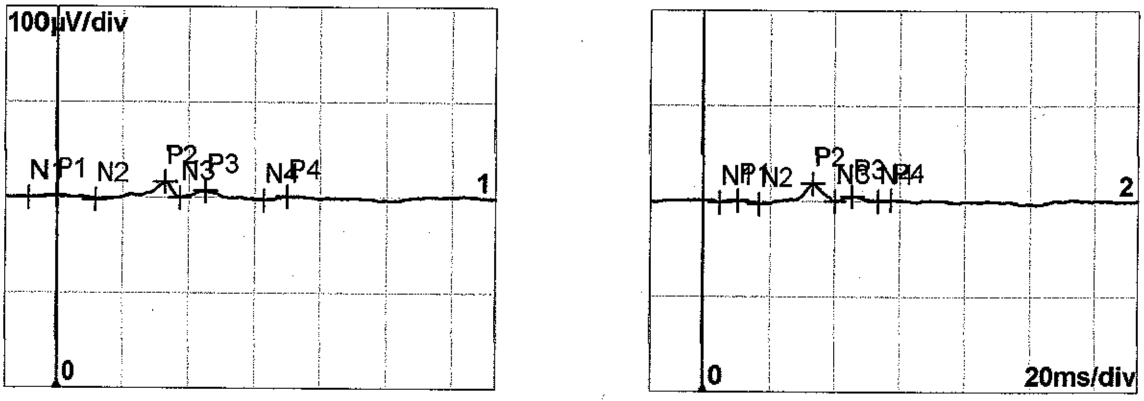

Channel	N1 [ms]	P1 [ms]	N2 [ms]	P2 [ms]	N3 [ms]	P3 [ms]	N4 [ms]	P4 [ms]	OS1	OS2	OS3	OS4	Total
1 R-1	-8	0	12	33	38	45	63	70	2.32µV	18.8µV	7.52µV	3.93µV	0.000V
2 L-2	5	11	17	34	41	46	54	58	2.44µV	21.8µV	4.91µV	1.29µV	0.000V

RETIport32

HEBEI EYE HOSPITAL
(No.399 Quanbeidong Rd.xingtai)

Patient: ZC-CEHN-3, CHen, 7/11/2023
Tested: 7/12/2023 9:34:17 PM
ID:
Sex/Age: M/0
Operator:
Electrode: NEEDLE Thread
Pupil Size: 2 dil.

Photopic 3.0 ERG

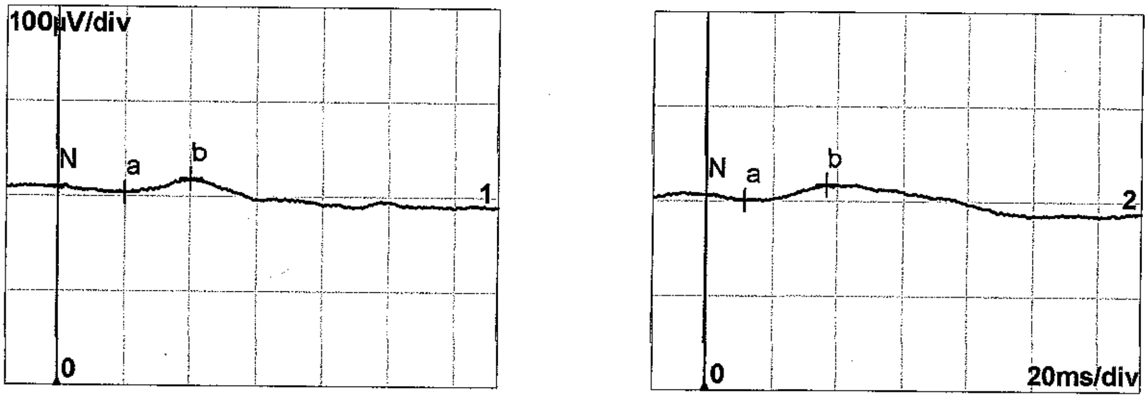

Channel	a [ms]	b [ms]	a-wave	b-wave
1 R-1	20	41	6.18µV	14.4µV
2 L-2	12	37	5.5µV	16.1µV

Photopic 3.0 flicker

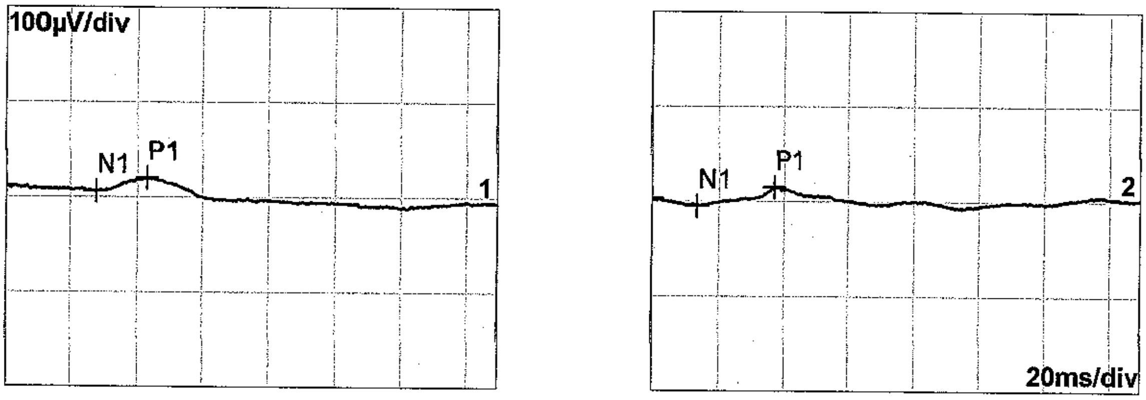

Channel	N1 [ms]	P1 [ms]	V1	N1-P1
1 R-1	28	43	5.83µV	14.2µV
2 L-2	14	37	7.84µV	20.1µV

RETIport32

HEBEI EYE HOSPITAL
(No.399 Quanbeidong Rd.xingtai)

Patient: ZC-CEHN-4, CHen, 7/11/2023
Tested: 7/12/2023 8:00:50 PM
ID:

Sex/Age: M/0
Operator:

Electrode: NEEDLE Thread
Pupil Size: 2 dil.

Scotopic 0.01 ERG

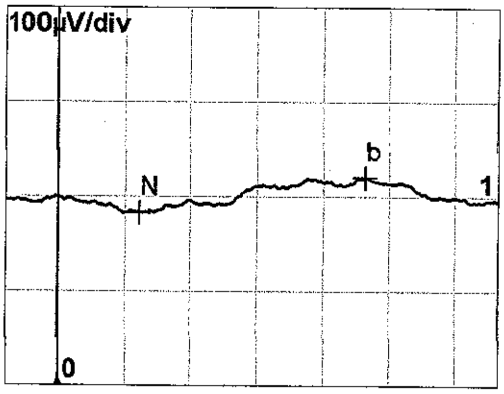

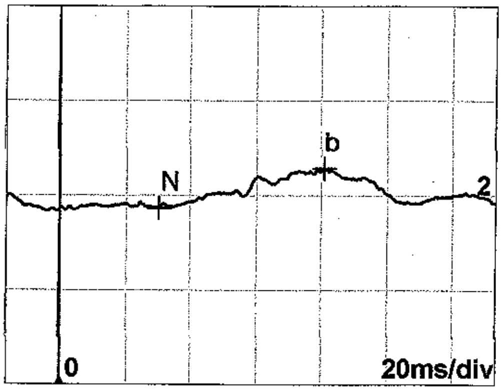

Channel	b [ms]	b-wave
1 R-1	93	37.5µV
2 L-2	81	42.2µV

Scotopic 3.0 ERG

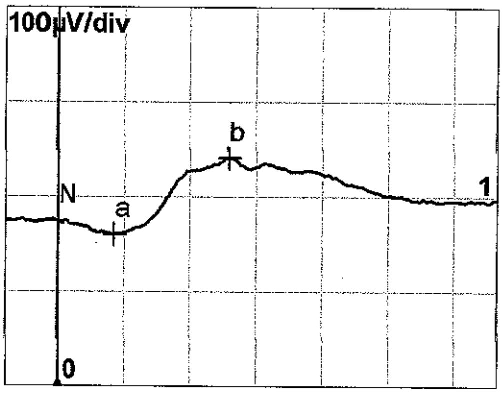

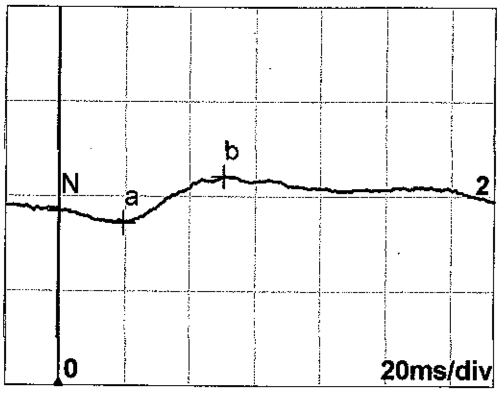

Channel	a [ms]	b [ms]	a-wave	b-wave	b/a
1 R-1	17	52	13.9µV	80.9µV	5.8V
2 L-2	20	51	12.4µV	48.6µV	3.9V

Scotopic 3.0 oscillatory potentials

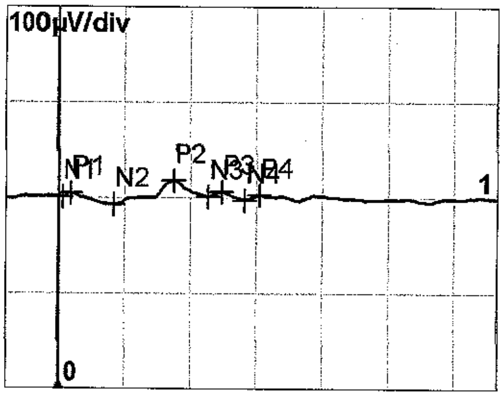

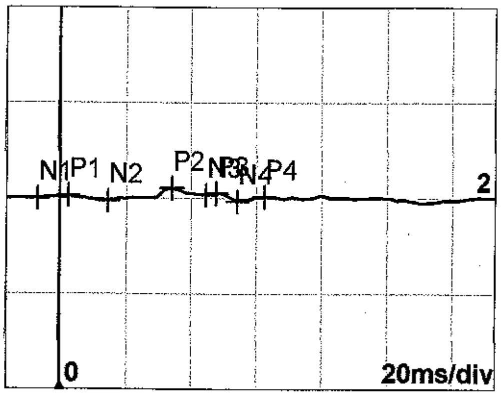

ChanneN1	[ms]P1	[ms]N2	[ms]P2	[ms]N3	[ms]P3	[ms]N4	[ms]P4	[ms]OS1		OS2	OS3	OS4	Total
1 R-1	1	4	17	35	46	50	57	61	3.88µV	25µV	4.71µV	5.57µV	0.000V
2 L-2	-7	3	15	35	45	48	55	63	2.99µV	13.2µV	1.67µV	4.81µV	0.000V

RETIport32	HEBEI EYE HOSPITAL<br>(No.399 Quanbeidong Rd.xingtai)
	Patient: ZC-CEHN-4, CHen, 7/11/2023<br>Tested: 7/12/2023 8:00:50 PM<br>ID:<br>Sex/Age: M/0<br>Operator:<br>Electrode: NEEDLE Thread<br>Pupil Size: 2 dil.

Photopic 3.0 ERG

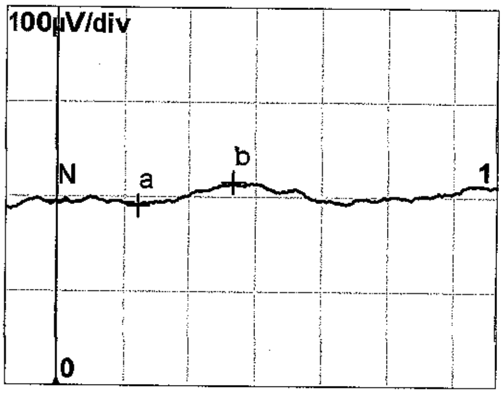

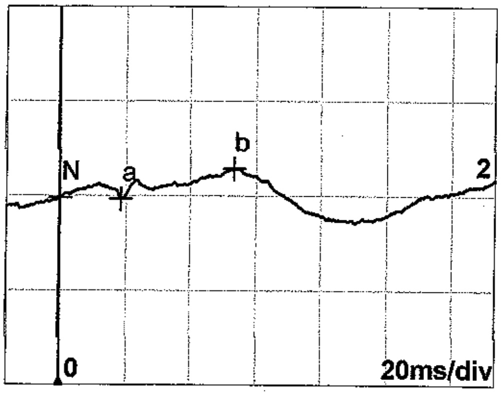

Channel	a [ms]	b [ms]	a-wave	b-wave
1 R-1	25	54	3.42µV	24.4µV
2 L-2	19	54	1.12µV	31.9µV

Photopic 3.0 flicker

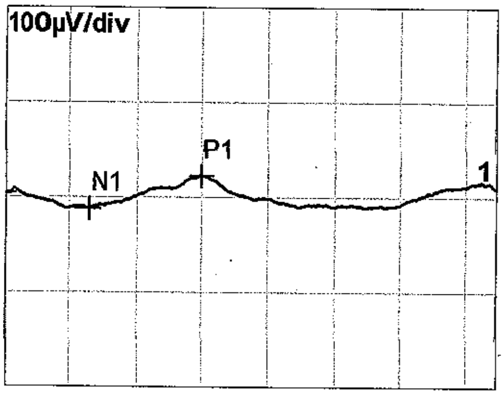

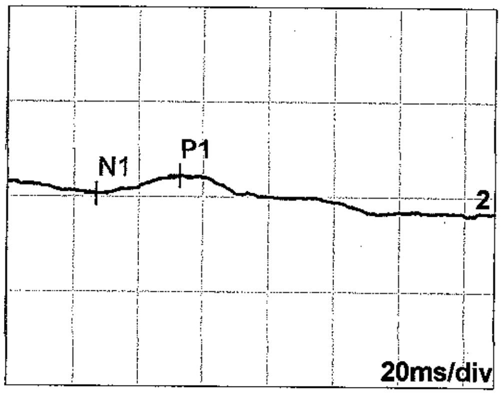

Channel	N1 [ms]	P1 [ms]	V1	N1-P1
1 R-1	26	60	17.2µV	34.6µV
2 L-2	27	53	12.8µV	18.8µV

HEBEI EYE HOSPITAL
(No.399 Quanbeidong Rd.xingtai)

Patient: ZC-CEHN-6, CHen, 7/11/2023
Tested: 7/12/2023 8:32:31 PM
ID:
Sex/Age: M/0
Operator:
Electrode: NEEDLE Thread
Pupil Size: 2 dil.

Scotopic 0.01 ERG

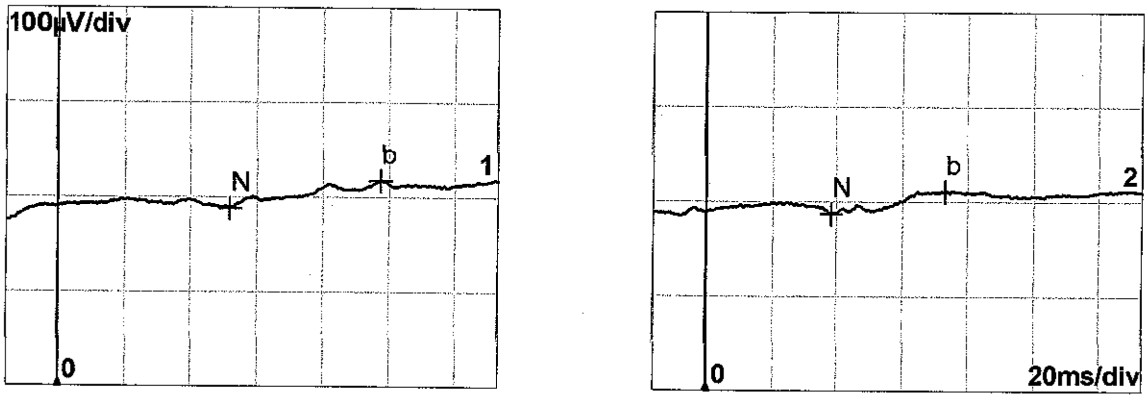

Channel	b [ms]	b-wave
1 R-1	98	29.2µV
2 L-2	73	23.6µV

Scotopic 3.0 ERG

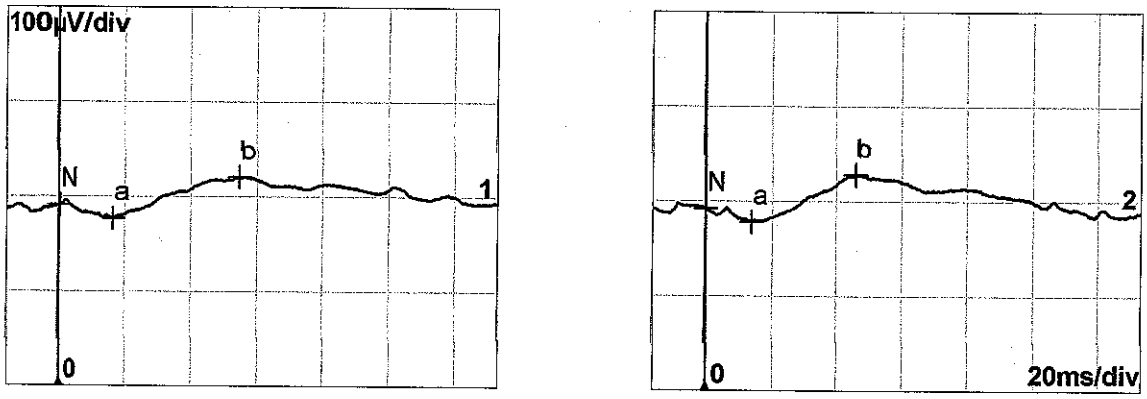

Channel	a [ms]	b [ms]	a-wave	b-wave	b/a
1 R-1	17	55	13.8µV	43.3µV	3.1V
2 L-2	14	46	13.8µV	49.7µV	3.6V

Scotopic 3.0 oscillatory potentials

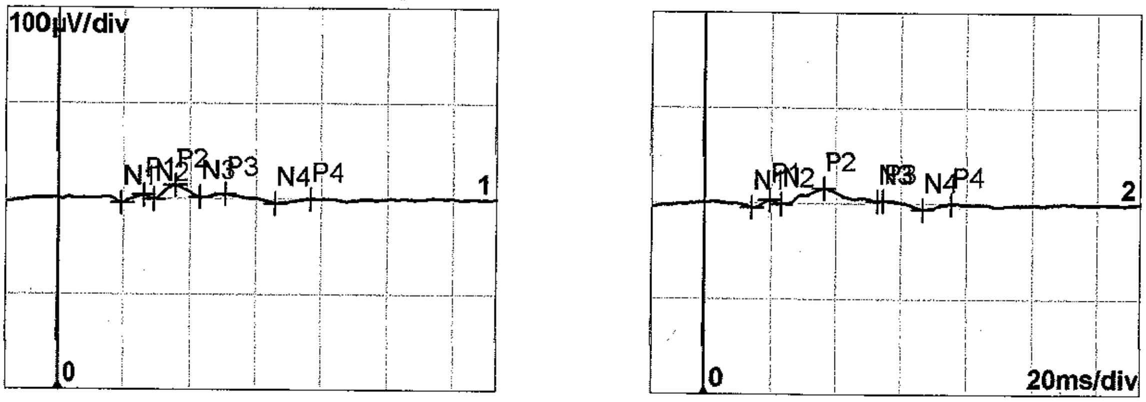

Channel	N1 [ms]	P1 [ms]	N2 [ms]	P2 [ms]	N3 [ms]	P3 [ms]	N4 [ms]	P4 [ms]	OS1	OS2	OS3	OS4	Total
1 R-1	20	26	29	36	43	51	66	77	8.25µV	14.6µV	3.52µV	4.32µV	0.000V
2 L-2	14	20	23	37	53	55	67	76	8.03µV	17.1µV	488nV	5.96µV	0.000V

Patient:	ZC-CEHN-6, CHen, 7/11/2023			Electrode:	NEEDLE Thread
Tested:	7/12/2023 8:32:31 PM	Sex/Age:	M/0	Pupil Size:	2 dil.
ID:		Operator:			

Photopic 3.0 ERG

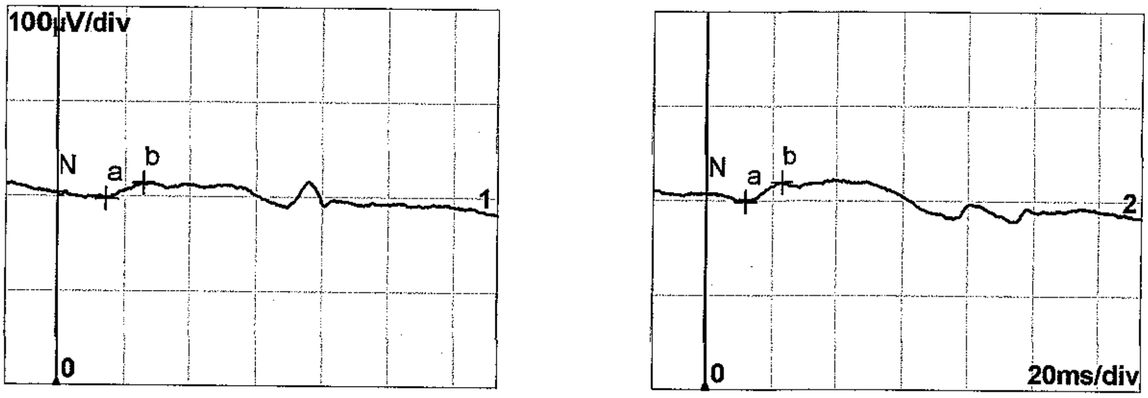

Channel	a [ms]	b [ms]	a-wave	b-wave
1 R-1	15	26	6.49µV	16.5µV
2 L-2	12	23	8.25µV	20.4µV

Photopic 3.0 flicker

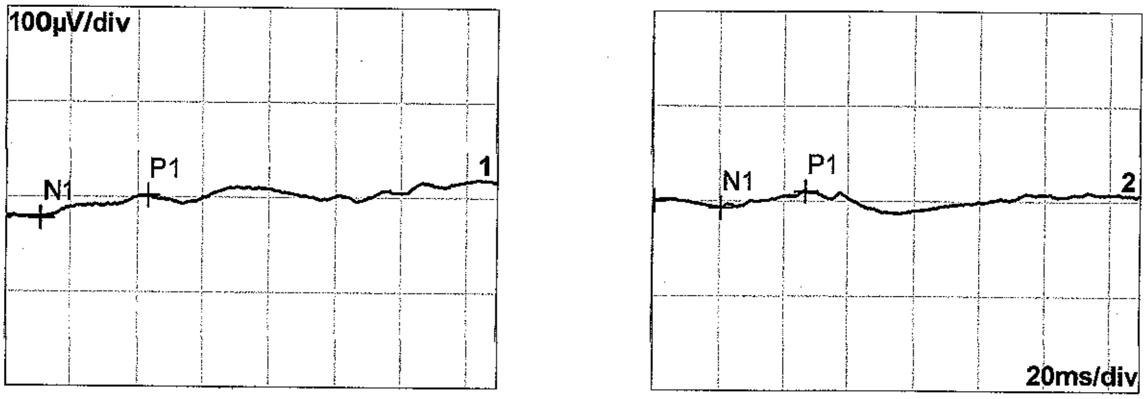

Channel	N1 [ms]	P1 [ms]	V1	N1-P1
1 R-1	11	44	2.6µV	24µV
2 L-2	21	48	7.15µV	17.4µV

RETIport32

HEBEI EYE HOSPITAL
(No.399 Quanbeidong Rd.xingtai)

Patient:	ZC-CEHN-7, CHen, 7/11/2023			Electrode:	NEEDLE Thread
Tested:	7/12/2023 7:27:14 PM	Sex/Age:	M/0	Pupil Size:	2 dil.
ID:		Operator:			

Scotopic 0.01 ERG

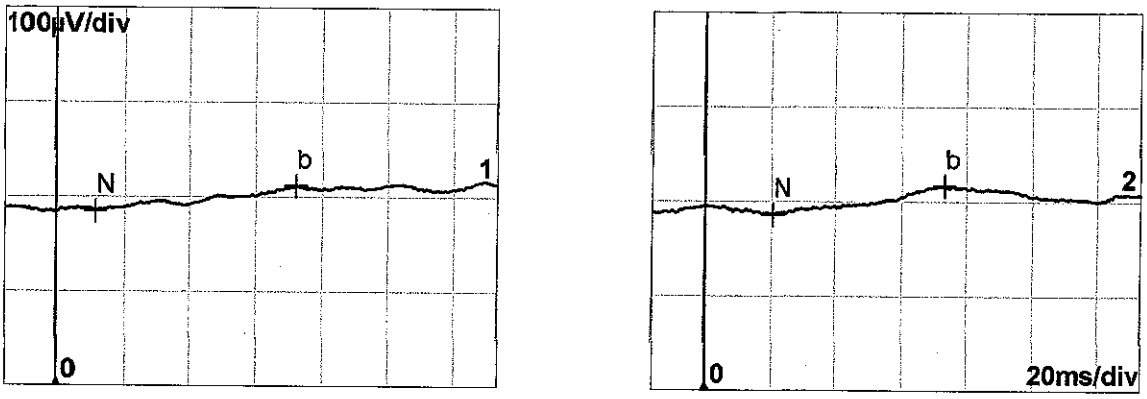

Channel	b [ms]	b-wave
1 R-1	73	26.5µV
2 L-2	74	31.1µV

Scotopic 3.0 ERG

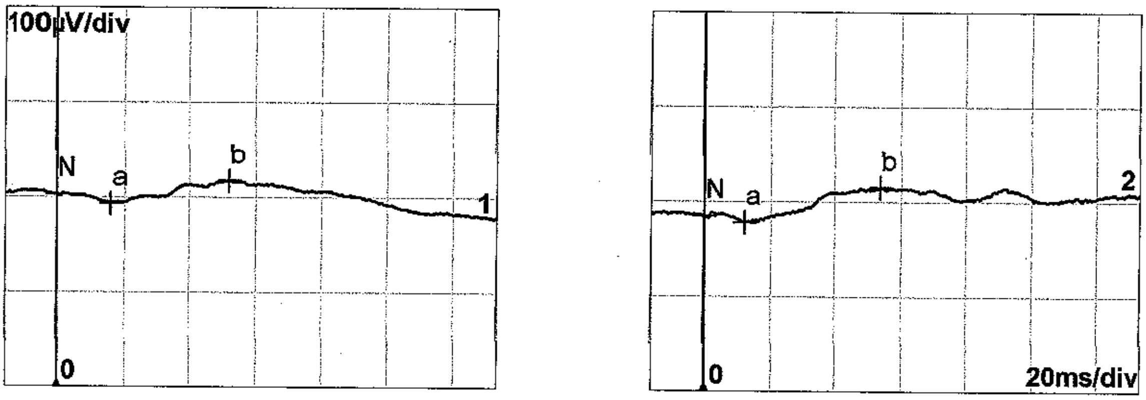

Channel	a [ms]	b [ms]	a-wave	b-wave	b/a
1 R-1	16	53	9.67µV	24.3µV	2.5V
2 L-2	12	54	6.81µV	36.7µV	5.4V

Scotopic 3.0 oscillatory potentials

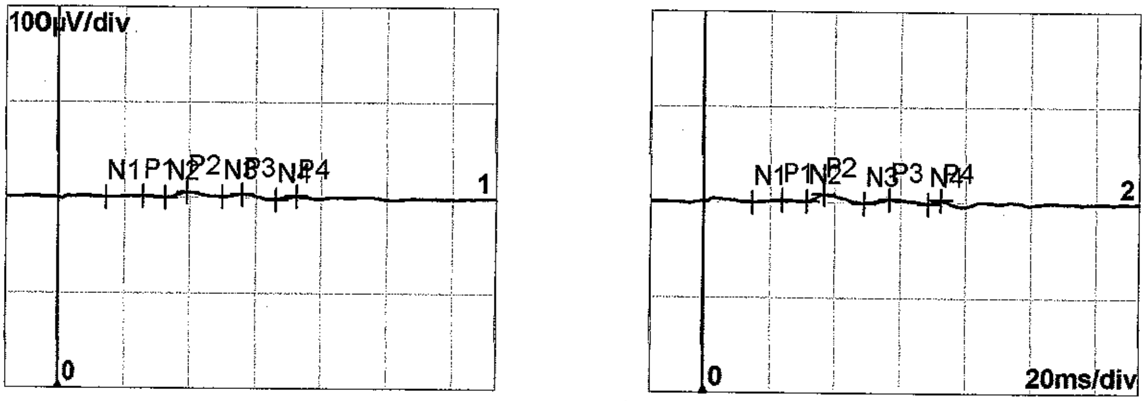

Channel	N1 [ms]	P1 [ms]	N2 [ms]	P2 [ms]	N3 [ms]	P3 [ms]	N4 [ms]	P4 [ms]	OS1	OS2	OS3	OS4	Total
1 R-1	15	26	33	40	50	56	67	73	1.56µV	5.32µV	2.7µV	3.42µV	0.000V
2 L-2	15	24	32	37	49	57	69	73	2.57µV	7.35µV	4.3µV	3.73µV	0.000V

RETIport32

HEBEI EYE HOSPITAL
(No.399 Quanbeidong Rd.xingtai)

Patient: ZC-CEHN-7, CHen, 7/11/2023
Tested: 7/12/2023 7:27:14 PM
ID:

Sex/Age: M/0
Operator:

Electrode: NEEDLE Thread
Pupil Size: 2 dil.

Photopic 3.0 ERG

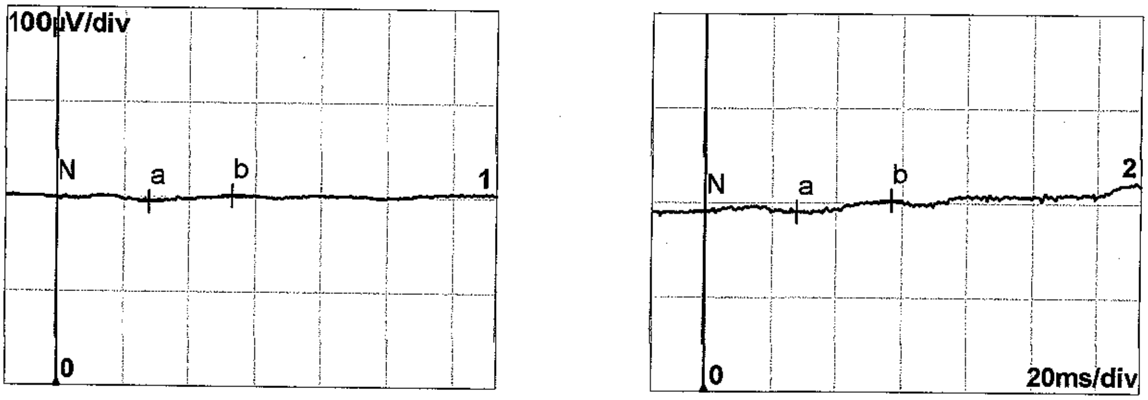

Channel	a [ms]	b [ms]	a-wave	b-wave
1 R-1	28	53	3.98µV	6.42µV
2 L-2	28	57	1.71µV	13.1µV

Photopic 3.0 flicker

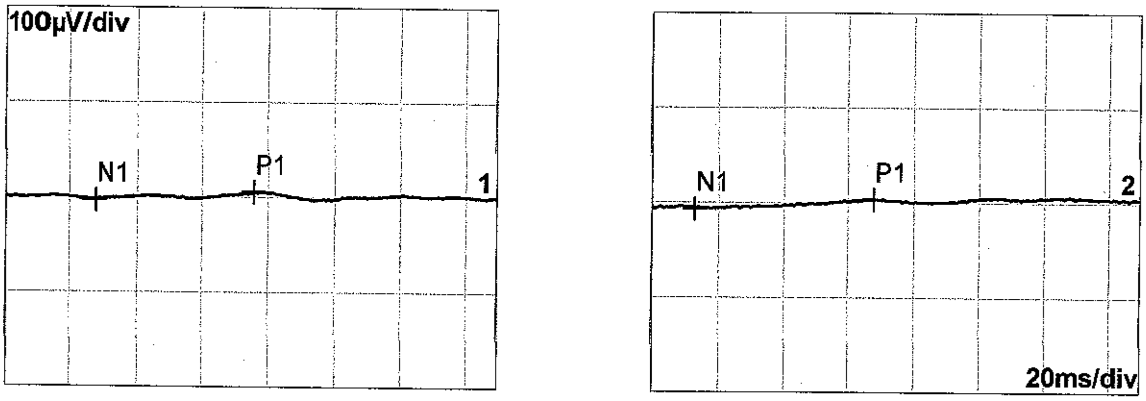

Channel	N1 [ms]	P1 [ms]	V1	N1-P1
1 R-1	28	76	3.33µV	8.89µV
2 L-2	13	68	1.14µV	10.2µV

RETIport32

HEBEI EYE HOSPITAL
(No.399 Quanbeidong Rd.xingtai)

Patient:	ZC-CEHN-8, CHen, 7/11/2023			Electrode:	NEEDLE Thread
Tested:	7/12/2023 10:02:42 PM	Sex/Age:	M/0	Pupil Size:	2 dil.
ID:		Operator:			

Photopic 3.0 ERG

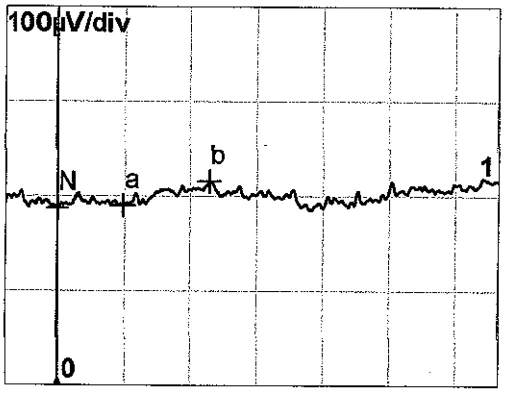

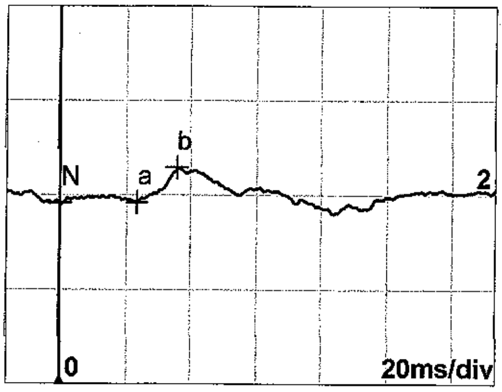

Channel	a [ms]	b [ms]	a-wave	b-wave
1 R-1	20	46	3.24µV	25.5µV
2 L-2	24	36	358nV	36.9µV

Photopic 3.0 flicker

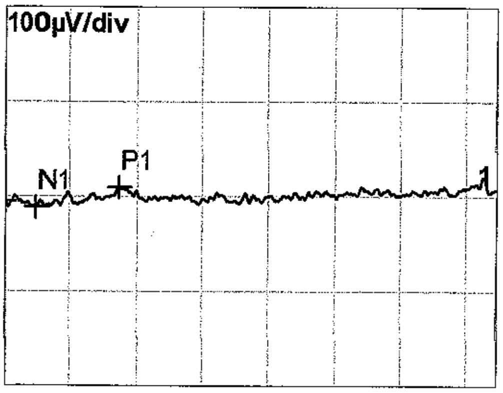

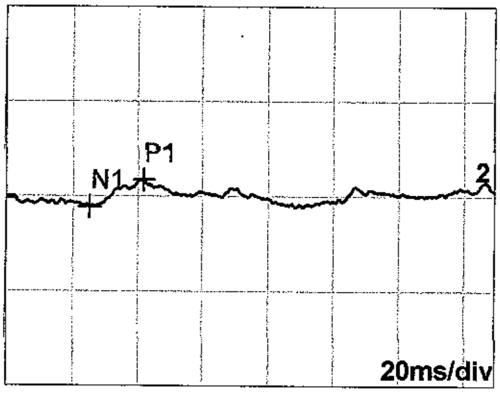

Channel	N1 [ms]	P1 [ms]	V1	N1-P1
1 R-1	10	35	6.54µV	21.1µV
2 L-2	26	42	9.28µV	28.5µV

RETIport32	HEBEI EYE HOSPITAL<br>(No.399 Quanbeidong Rd.xingtai)		
	Patient: ZC-CEHN-8, CHen, 7/11/2023<br>Tested: 7/12/2023 10:02:42 PM<br>ID:	Sex/Age: M/0<br>Operator:	Electrode: NEEDLE Thread<br>Pupil Size: 2 dil.

Scotopic 0.01 ERG

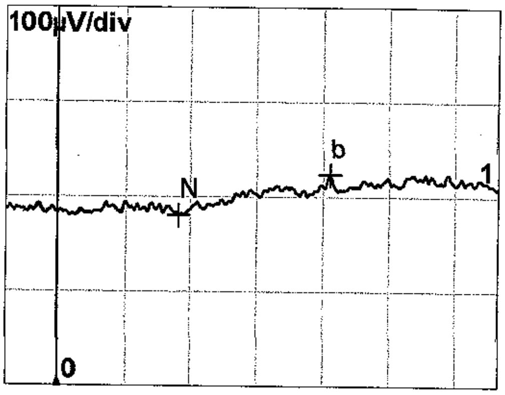

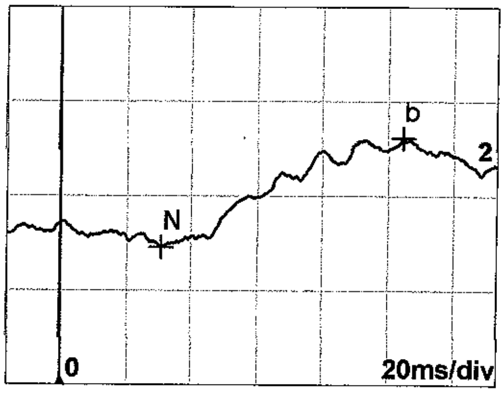

Channel	b [ms]	b-wave
1 R-1	82	42.8µV
2 L-2	105	116µV

Scotopic 3.0 ERG

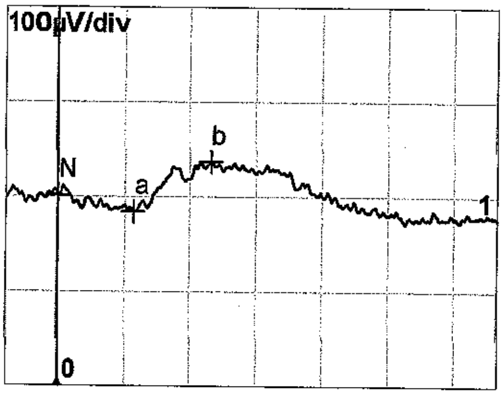

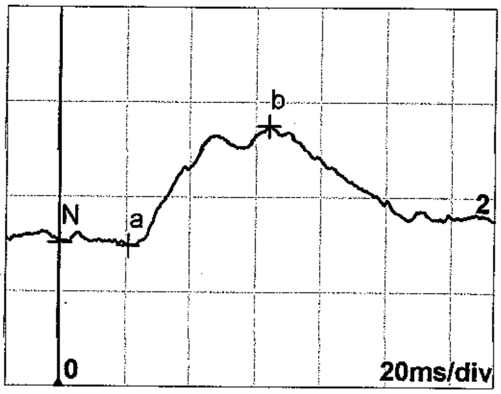

Channel	a [ms]	b [ms]	a-wave	b-wave	b/a
1 R-1	23	47	16.8µV	52.8µV	3.1V
2 L-2	21	65	3.69µV	127µV	34V

Scotopic 3.0 oscillatory potentials

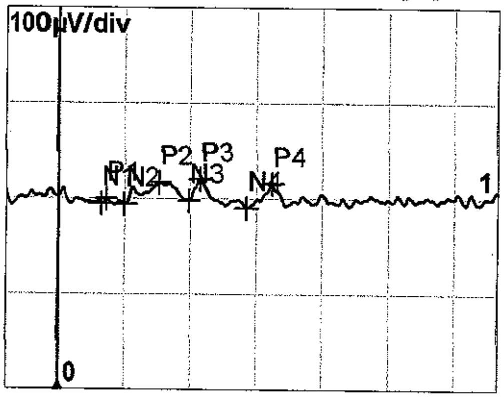

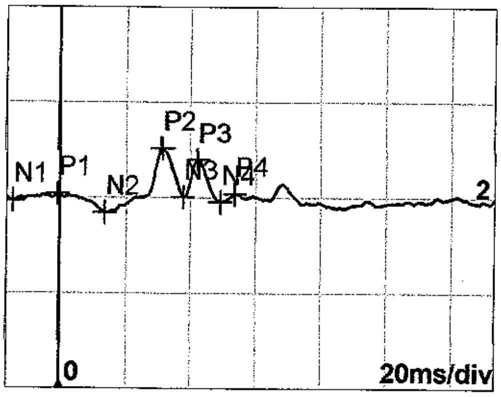

Channel	N1 [ms]	P1 [ms]	N2 [ms]	P2 [ms]	N3 [ms]	P3 [ms]	N4 [ms]	P4 [ms]	OS1	OS2	OS3	OS4	Total
1 R-1	13	15	20	31	40	43	58	65	4.98µV	23.2µV	22.4µV	25.7µV	0.000V
2 L-2	-14	-1	14	32	38	43	50	54	7.28µV	67.8µV	38.8µV	8.54µV	0.000V

RETIport32	HEBEI EYE HOSPITAL<br>(No.399 Quanbeidong Rd.xingtai)		
	Patient: ZC-CEHN-13, CHen, 7/11/2023<br>Tested: 7/12/2023 9:09:15 PM<br>ID:	Sex/Age: M/0<br>Operator:	Electrode: NEEDLE Thread<br>Pupil Size: 2 dil.

Scotopic 0.01 ERG

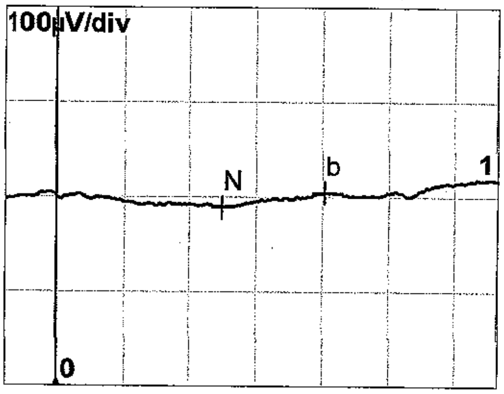

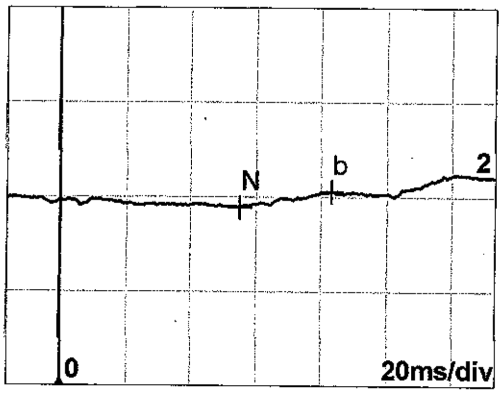

Channel	b [ms]	b-wave
1 R-1	81	15.9µV
2 L-2	84	16.5µV

Scotopic 3.0 ERG

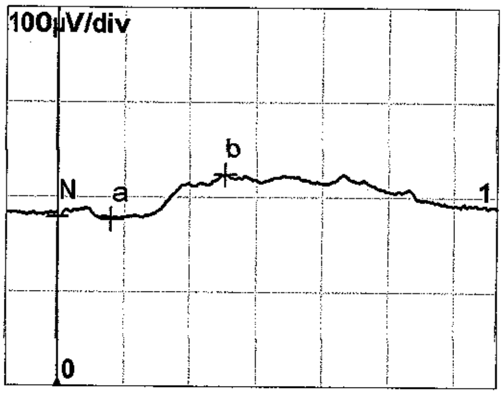

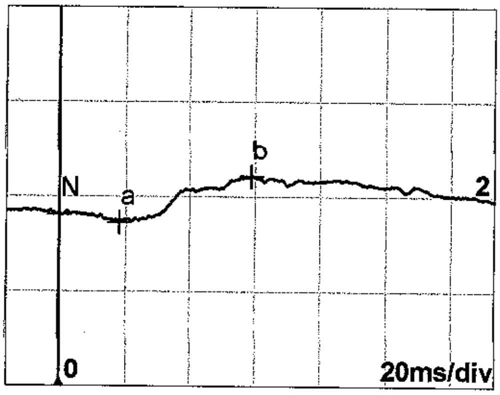

Channel	a [ms]	b [ms]	a-wave	b-wave	b/a
1 R-1	17	51	2.17µV	47.1µV	22V
2 L-2	18	59	8.25µV	48.8µV	5.9V

Scotopic 3.0 oscillatory potentials

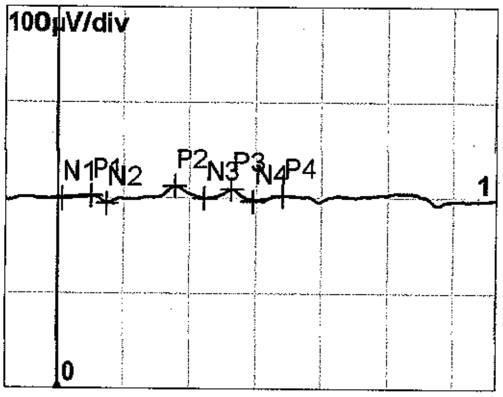

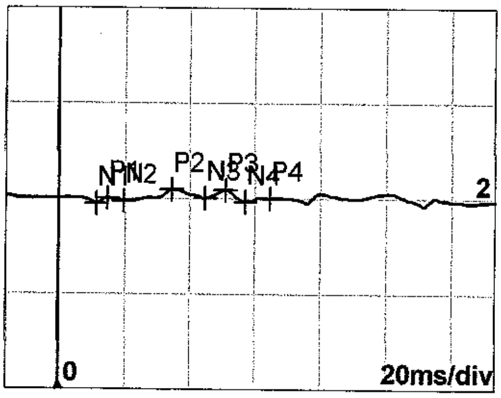

Channel	N1 [ms]	P1 [ms]	N2 [ms]	P2 [ms]	N3 [ms]	P3 [ms]	N4 [ms]	P4 [ms]	OS1	OS2	OS3	OS4	Total
1 R-1	1	10	15	36	45	53	60	69	3.1µV	18.2µV	9.5µV	5.83µV	0.000V
2 L-2	11	15	20	35	45	51	58	65	5.59µV	12.7µV	8.42µV	4.13µV	0.000V

RETIport32	HEBEI EYE HOSPITAL<br>(No.399 Quanbeidong Rd.xingtai)
	Patient: **ZC-CEHN-13, CHen, 7/11/2023**<br>Tested: 7/12/2023 9:09:15 PM<br>ID:<br>Sex/Age: M/0<br>Operator:<br>Electrode: NEEDLE Thread<br>Pupil Size: 2 dil.

Photopic 3.0 ERG

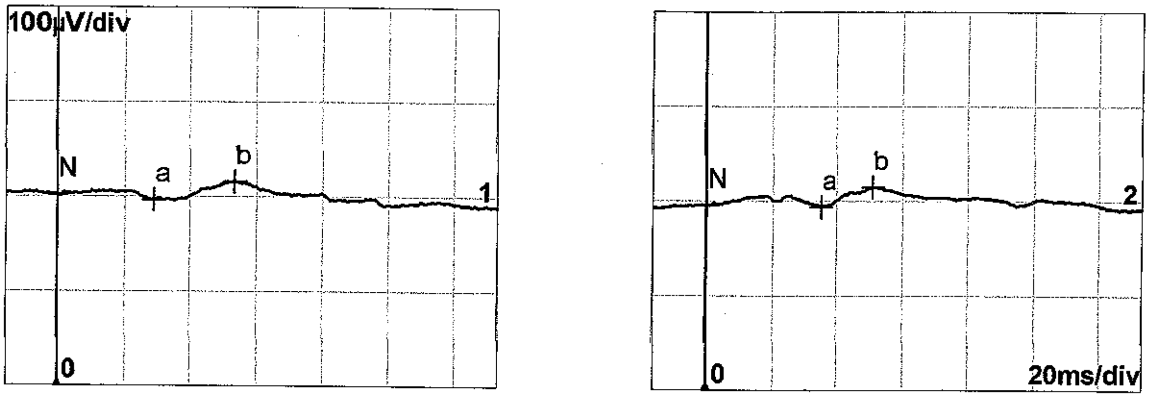

Channel	a [ms]	b [ms]	a-wave	b-wave
1 R-1	29	54	5.12µV	19µV
2 L-2	36	51	1.3µV	20.5µV

Photopic 3.0 flicker

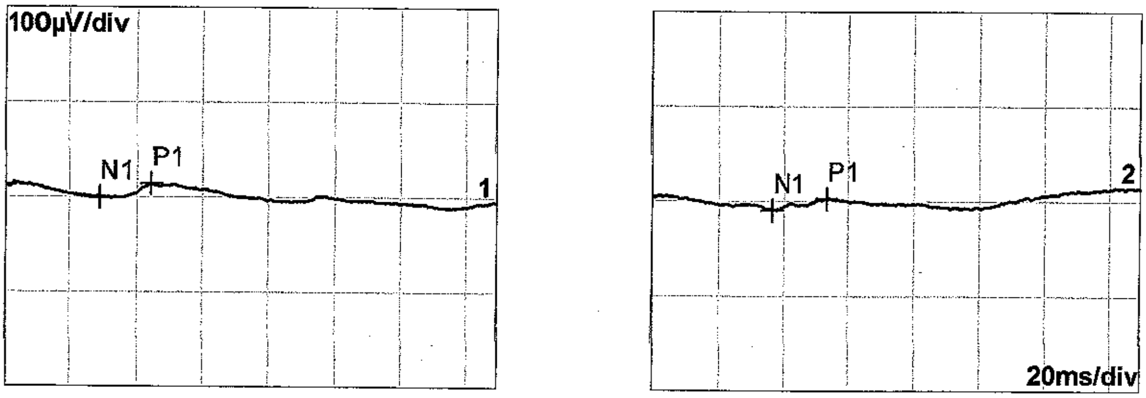

Channel	N1 [ms]	P1 [ms]	V1	N1-P1
1 R-1	29	45	13.7µV	15µV
2 L-2	37	54	14.4µV	12.6µV

RETIport32	HEBEI EYE HOSPITAL<br>(No.399 Quanbeidong Rd.xingtai)		
	Patient: ZC-CEHN-1, CHen, 7/11/2023<br>Tested: 7/20/2023 10:43:45 PM<br>ID:	Sex/Age: M/0<br>Operator:	Electrode: NEEDLE Thread<br>Pupil Size: 2 dil.

Scotopic 0.01 ERG

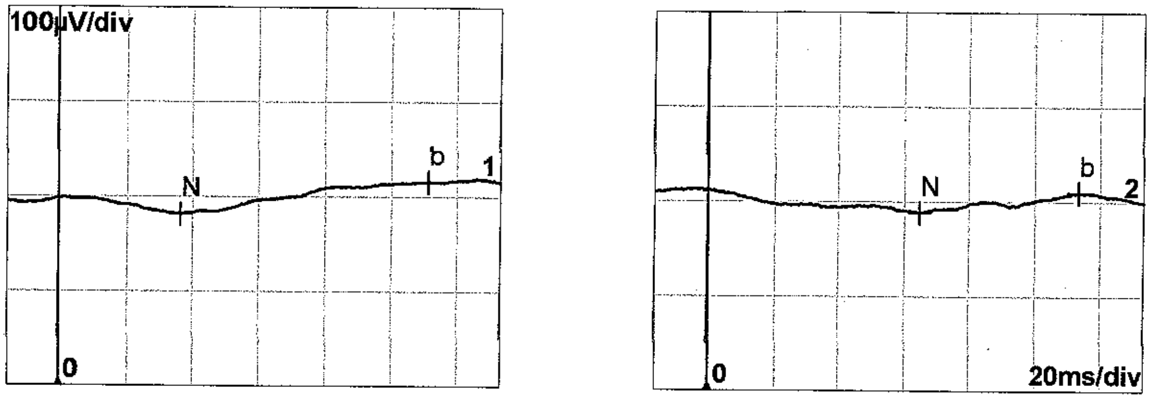

Channel	b [ms]	b-wave
1 R-1	112	34.2µV
2 L-2	114	21µV

Scotopic 3.0 ERG

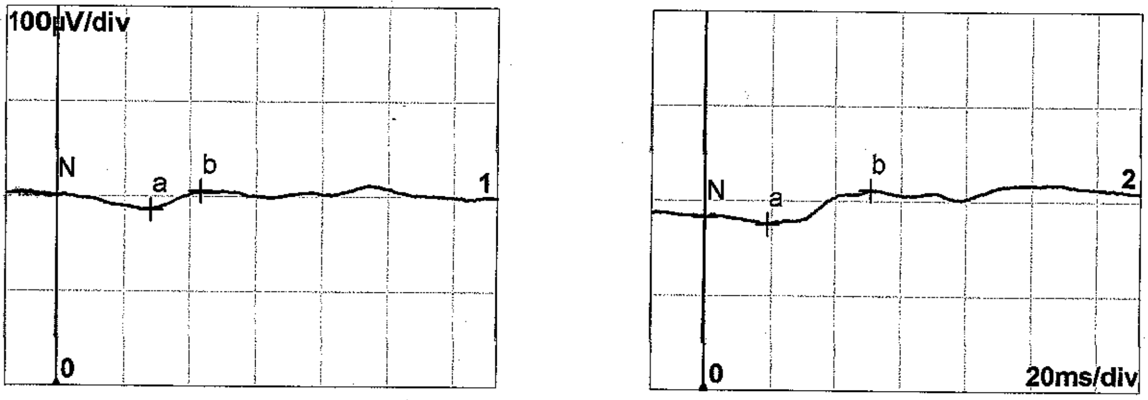

Channel	a [ms]	b [ms]	a-wave	b-wave	b/a
1 R-1	28	43	15.2µV	19.5µV	1.3V
2 L-2	19	51	7.35µV	36.1µV	4.9V

Scotopic 3.0 oscillatory potentials

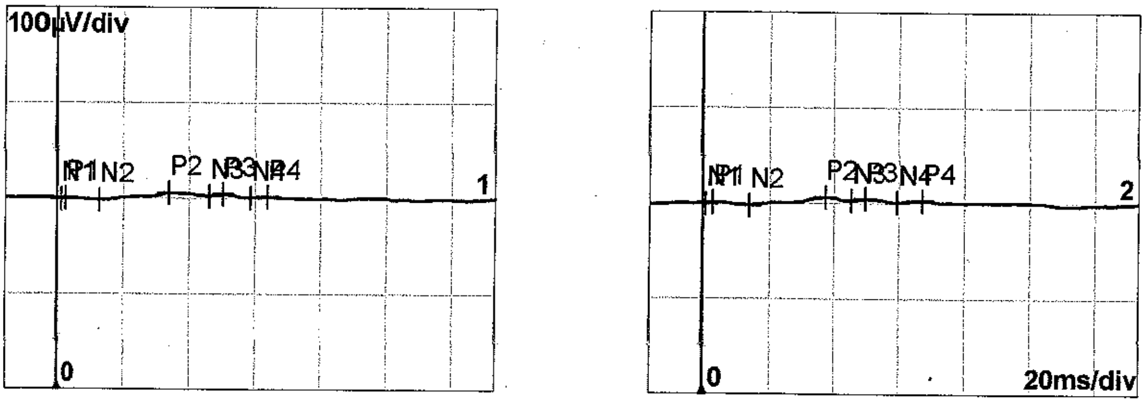

Channe	N1 [ms]	P1 [ms]	N2 [ms]	P2 [ms]	N3 [ms]	P3 [ms]	N4 [ms]	P4 [ms]	OS1	OS2	OS3	OS4	Total
1 R-1	1	3	13	34	46	50	59	64	1.56µV	7.67µV	2.29µV	1.09µV	0.000V
2 L-2	1	3	14	38	46	50	60	68	1.79µV	9.12µV	1.73µV	2µV	0.000V

RETIport32

HEBEI EYE HOSPITAL
(No.399 Quanbeidong Rd.xingtai)

Patient: ZC-CEHN-1, CHen, 7/11/2023
Tested: 7/20/2023 10:43:45 PM
ID:

Sex/Age: M/0
Operator:

Electrode: NEEDLE Thread
Pupil Size: 2 dil.

Photopic 3.0 ERG

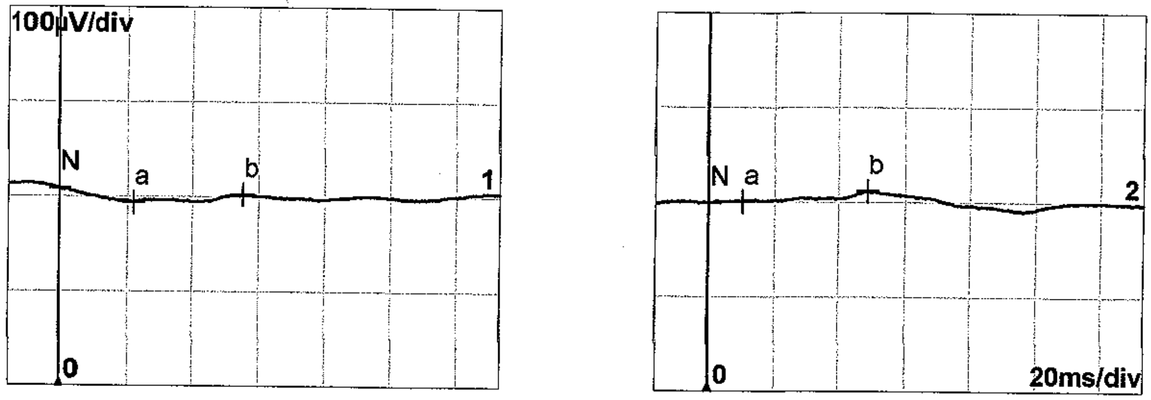

Channel	a [ms]	b [ms]	a-wave	b-wave
1 R-1	22	55	14.7µV	8.16µV
2 L-2	11	49	2.05µV	11.1µV

Photopic 3.0 flicker

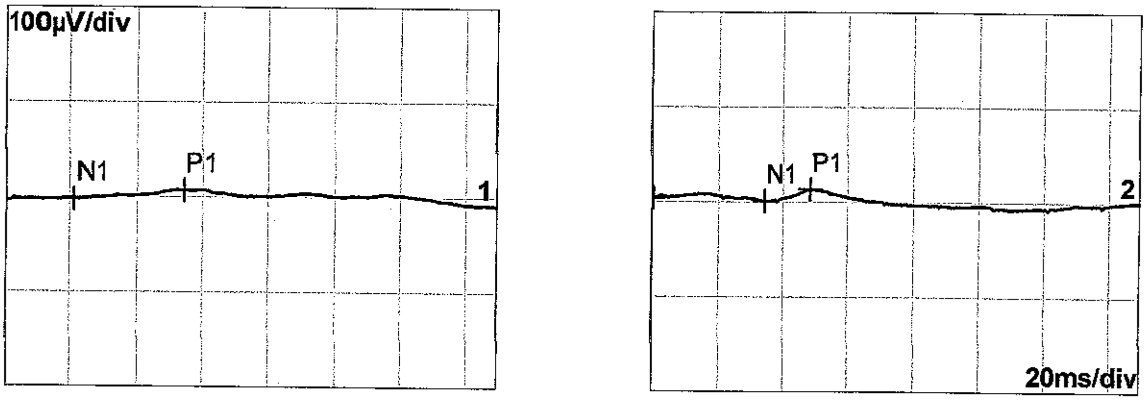

Channel	N1 [ms]	P1 [ms]	V1	N1-P1
1 R-1	22	55	916nV	9.52µV
2 L-2	35	49	2.78µV	12.3µV

RETIport32	HEBEI EYE HOSPITAL<br>(No.399 Quanbeidong Rd.xingtai)

Patient: ZC-CEHN-3, CHen, 7/11/2023
Tested: 7/20/2023 7:30:54 PM
ID:
Sex/Age: M/0
Operator:
Electrode: NEEDLE Thread
Pupil Size: 2 dil.

Scotopic 0.01 ERG

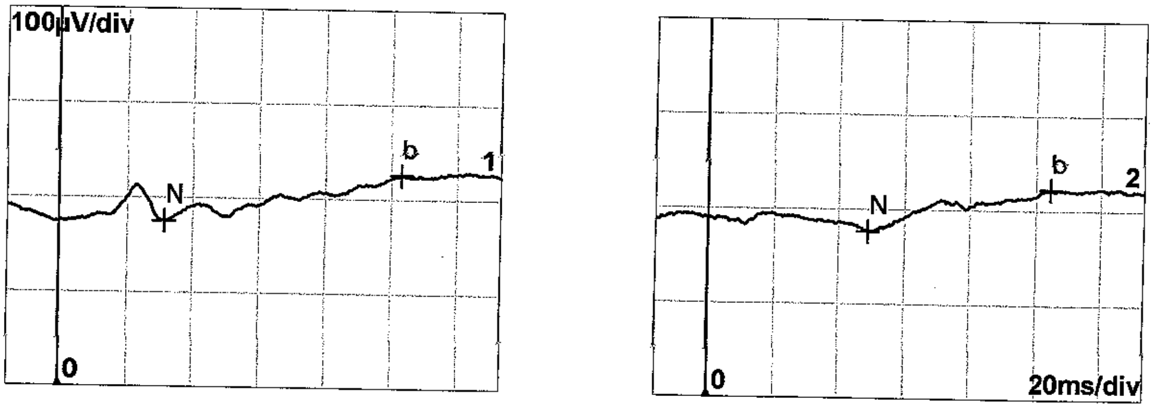

Channel	b [ms]	b-wave
1 R-1	104	50µV
2 L-2	105	45.9µV

Scotopic 3.0 ERG

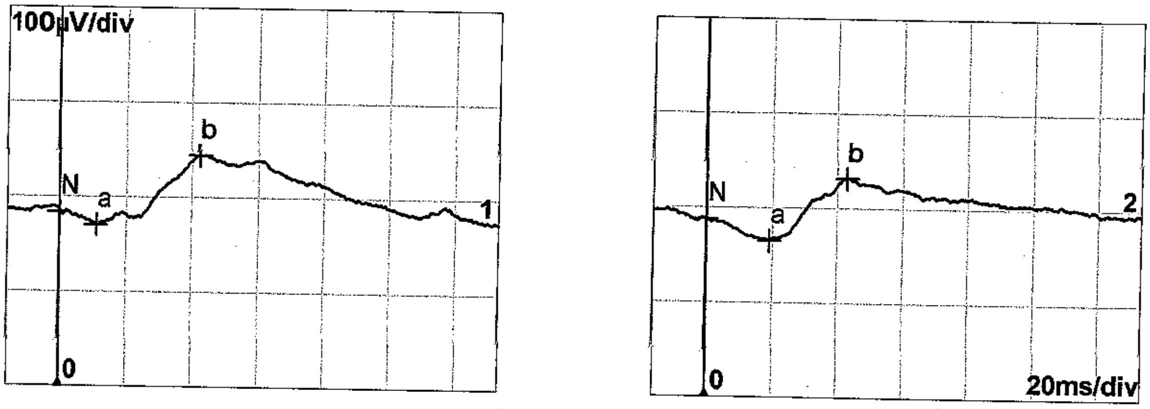

Channel	a [ms]	b [ms]	a-wave	b-wave	b/a
1 R-1	11	43	13.4µV	73.9µV	5.5V
2 L-2	19	43	22.1µV	66.1µV	3V

Scotopic 3.0 oscillatory potentials

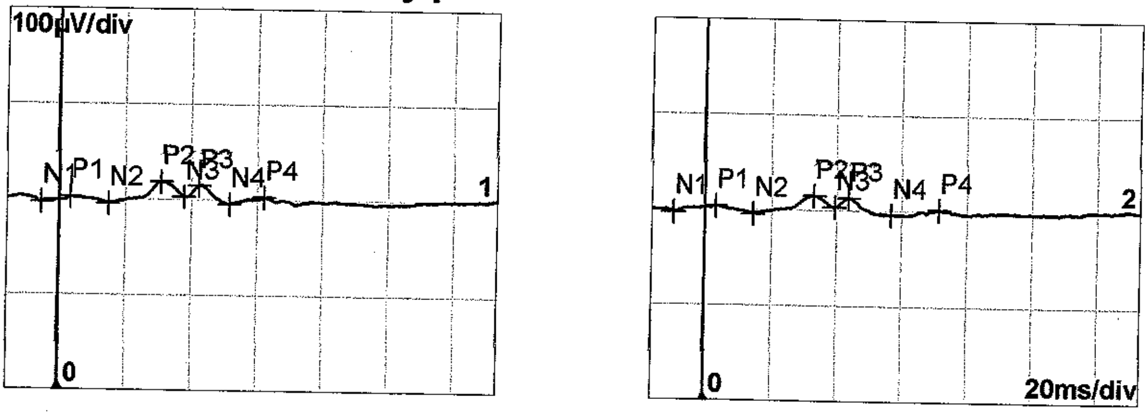

Channel	N1 [ms]	P1 [ms]	N2 [ms]	P2 [ms]	N3 [ms]	P3 [ms]	N4 [ms]	P4 [ms]	OS1	OS2	OS3	OS4	Total
1 R-1	-5	4	15	31	38	43	52	63	5.66µV	22.7µV	11.5µV	7.28µV	0.000V
2 L-2	-10	3	15	34	40	44	57	72	6.91µV	19.7µV	9.33µV	6.27µV	0.000V

HEBEI EYE HOSPITAL
(No.399 Quanbeidong Rd.xingtai)

Patient:	ZC-CEHN-3, CHen, 7/11/2023		Electrode: NEEDLE Thread
Tested:	7/20/2023 7:30:54 PM	Sex/Age: M/0	Pupil Size: 2 dil.
ID:		Operator:	

Photopic 3.0 ERG

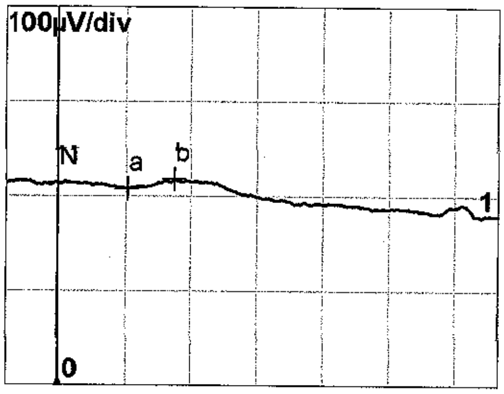

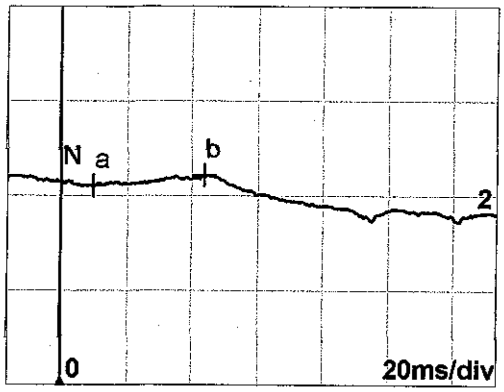

Channel	a [ms]	b [ms]	a-wave	b-wave
1 R-1	21	35	6.2µV	10.4µV
2 L-2	10	44	4µV	10.6µV

Photopic 3.0 flicker

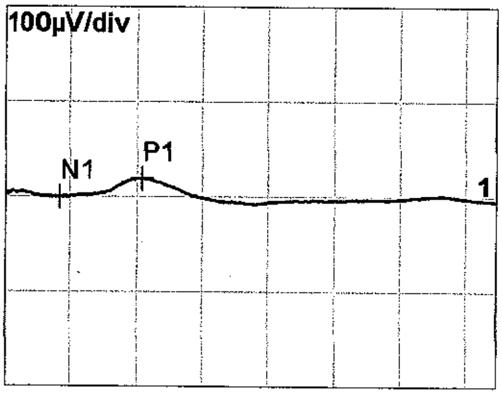

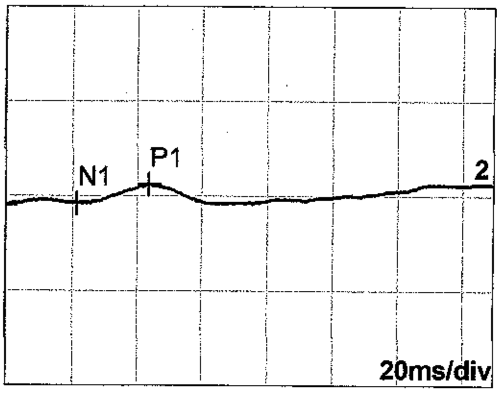

Channel	N1 [ms]	P1 [ms]	V1	N1-P1
1 R-1	17	42	5.19µV	19.4µV
2 L-2	22	44	1.77µV	20.2µV

RETIport32	HEBEI EYE HOSPITAL<br>(No.399 Quanbeidong Rd.xingtai)

Patient: ZC-CEHN-4, CHen, 7/11/2023
Tested: 7/20/2023 9:55:05 PM
ID:
Sex/Age: M/0
Operator:
Electrode: NEEDLE Thread
Pupil Size: 2 dil.

Scotopic 0.01 ERG

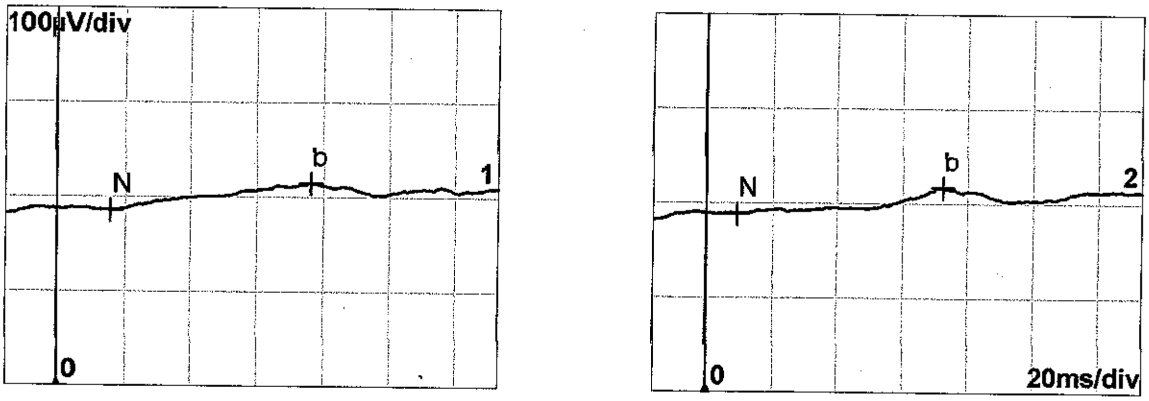

Channel	b [ms]	b-wave
1 R-1	77	29.4µV
2 L-2	73	28.5µV

Scotopic 3.0 ERG

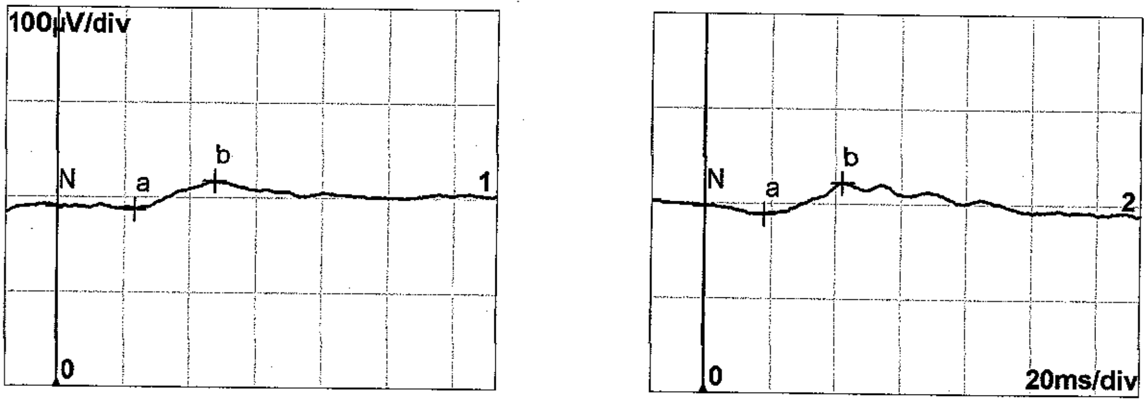

Channel	a [ms]	b [ms]	a-wave	b-wave	b/a
1 R-1	23	48	2.71µV	29.9µV	11V
2 L-2	18	43	8.67µV	33.3µV	3.8V

Scotopic 3.0 oscillatory potentials

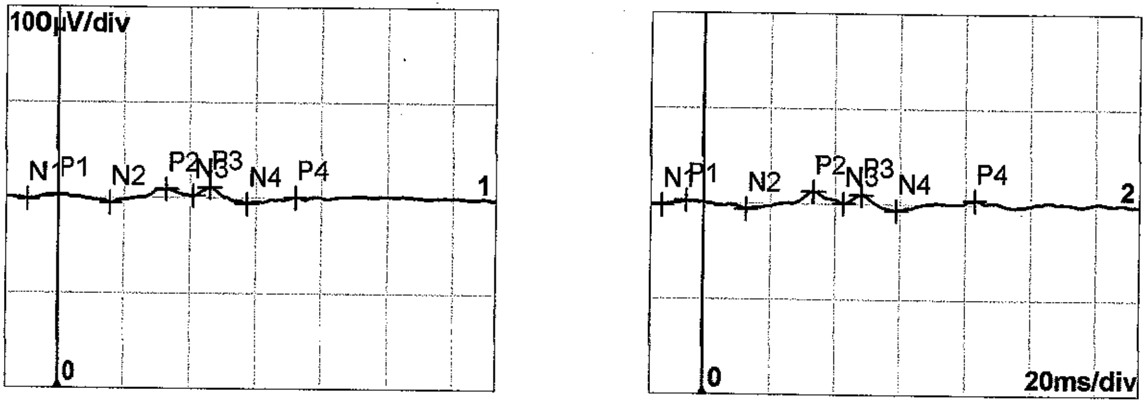

Channel	N1 [ms]	P1 [ms]	N2 [ms]	P2 [ms]	N3 [ms]	P3 [ms]	N4 [ms]	P4 [ms]	OS1	OS2	OS3	OS4	Total
1 R-1	-9	0	16	33	41	46	58	73	5.1µV	14.9µV	8.76µV	6.86µV	0.000V
2 L-2	-13	-5	13	34	43	48	59	83	5.1µV	18.6µV	8.98µV	11.1µV	0.000V

Patient: ZC-CEHN-4, CHen, 7/11/2023
Tested: 7/20/2023 9:55:05 PM
ID:
Sex/Age: M/0
Operator:
Electrode: NEEDLE Thread
Pupil Size: 2 dil.

Photopic 3.0 ERG

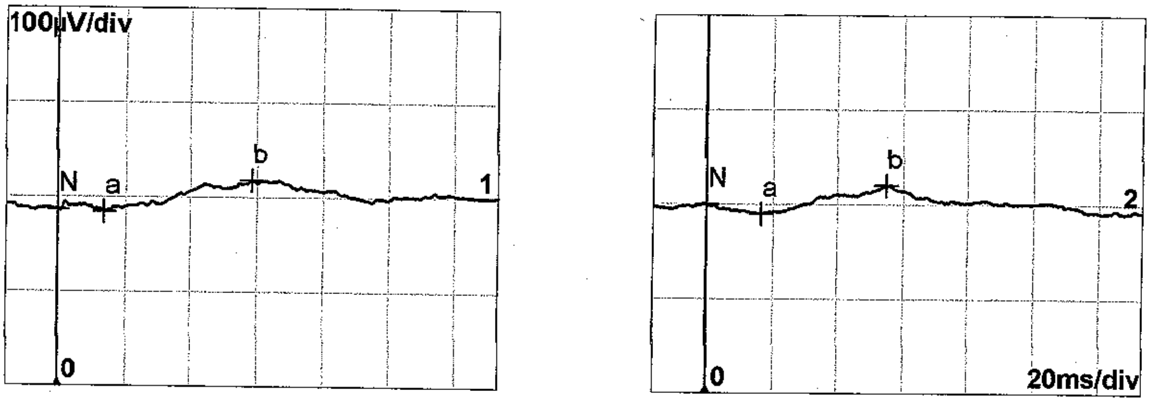

Channel	a [ms]	b [ms]	a-wave	b-wave
1 R-1	14	59	2.05µV	33.4µV
2 L-2	17	55	10.3µV	30.7µV

Photopic 3.0 flicker

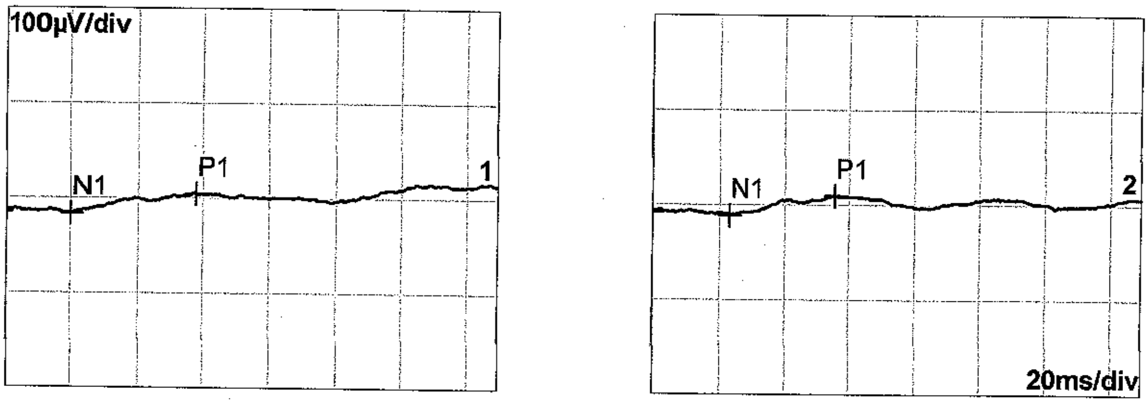

Channel	N1 [ms]	P1 [ms]	V1	N1-P1
1 R-1	21	58	4.95µV	20.9µV
2 L-2	23	56	3.26µV	20.4µV

RETIport32

HEBEI EYE HOSPITAL
(No.399 Quanbeidong Rd.xingtai)

Patient: ZC-CEHN-6, CHen, 7/11/2023
Tested: 7/20/2023 8:52:37 PM
ID:
Sex/Age: M/0
Operator:
Electrode: NEEDLE Thread
Pupil Size: 2 dil.

Scotopic 0.01 ERG

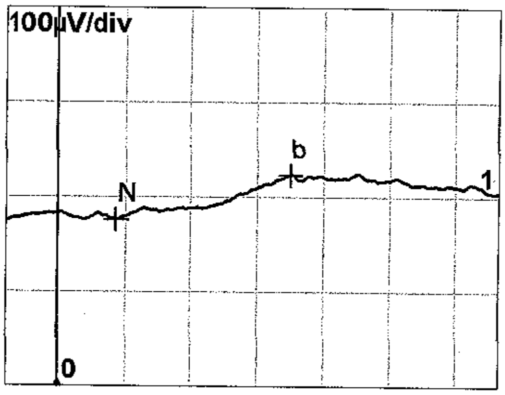

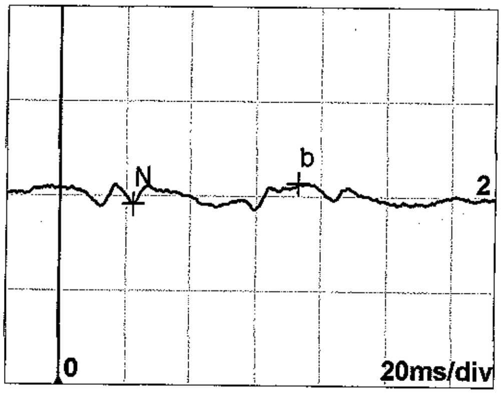

Channel	b [ms]	b-wave
1 R-1	70	47.3µV
2 L-2	73	21.7µV

Scotopic 3.0 ERG

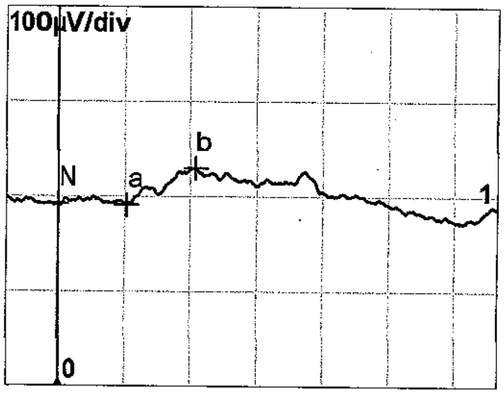

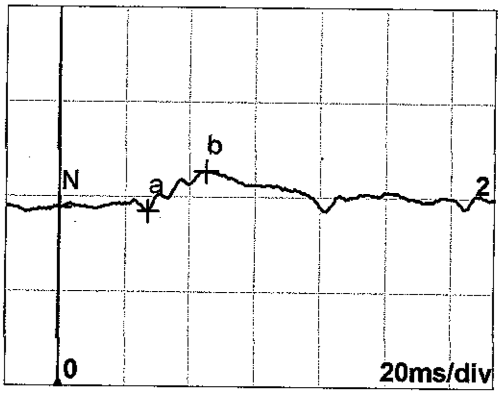

Channel	a [ms]	b [ms]	a-wave	b-wave	b/a
1 R-1	21	42	1.17µV	38.8µV	33V
2 L-2	28	45	3.25µV	41.9µV	13V

Scotopic 3.0 oscillatory potentials

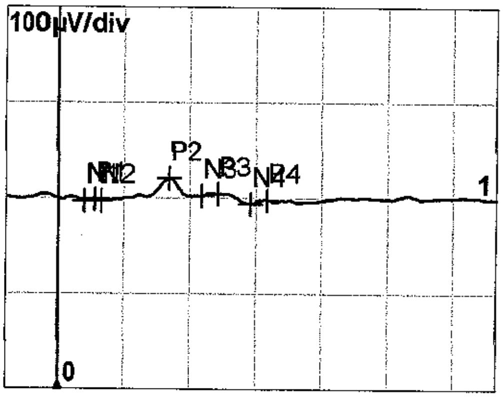

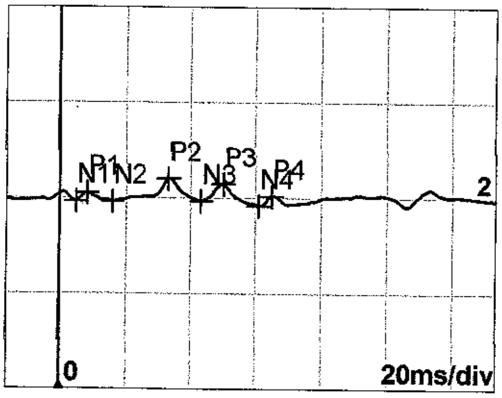

Channel	N1 [ms]	P1 [ms]	N2 [ms]	P2 [ms]	N3 [ms]	P3 [ms]	N4 [ms]	P4 [ms]	OS1	OS2	OS3	OS4	Total
1 R-1	9	11	13	34	44	49	59	64	1.97µV	24µV	2.54µV	4.1µV	0.000V
2 L-2	5	9	16	34	44	50	61	65	8.74µV	24.2µV	17.5µV	9.9µV	0.000V

RETIport32

HEBEI EYE HOSPITAL
(No.399 Quanbeidong Rd.xingtai)

Patient: ZC-CEHN-6, CHen, 7/11/2023
Tested: 7/20/2023 8:52:37 PM
ID:
Sex/Age: M/0
Operator:
Electrode: NEEDLE Thread
Pupil Size: 2 dil.

Photopic 3.0 ERG

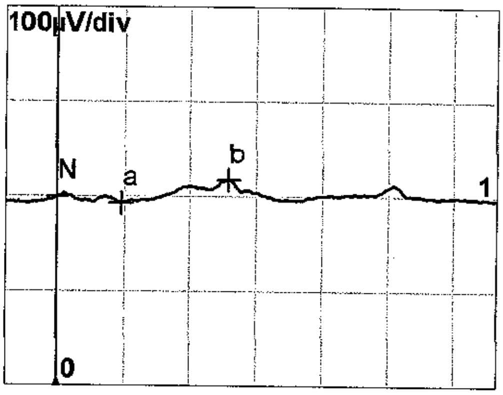

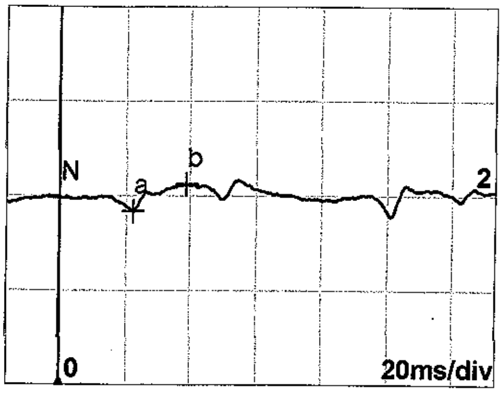

Channel	a [ms]	b [ms]	a-wave	b-wave
1 R-1	19	52	6.77µV	24.8µV
2 L-2	23	39	14.1µV	28.2µV

Photopic 3.0 flicker

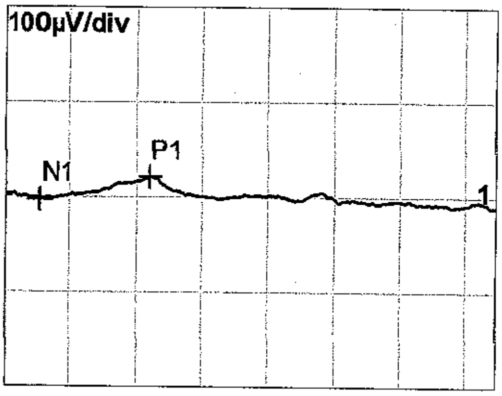

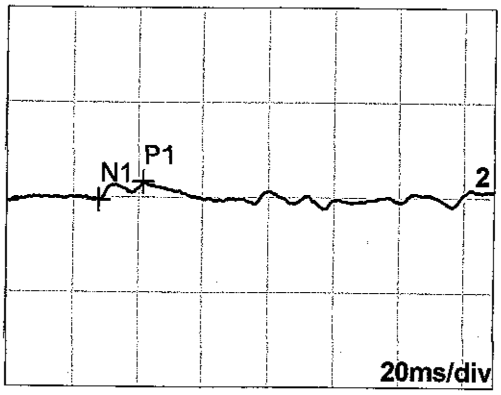

Channel	N1 [ms]	P1 [ms]	V1	N1-P1
1 R-1	11	45	6.78µV	24.4µV
2 L-2	28	42	2.11µV	18.7µV

RETIport32

HEBEI EYE HOSPITAL
(No.399 Quanbeidong Rd.xingtai)

Patient:	ZC-CEHN-7, CHen, 7/11/2023		Electrode: NEEDLE Thread
Tested:	7/20/2023 9:25:25 PM	Sex/Age: M/0	Pupil Size: 2 dil.
ID:		Operator:	

Scotopic 0.01 ERG

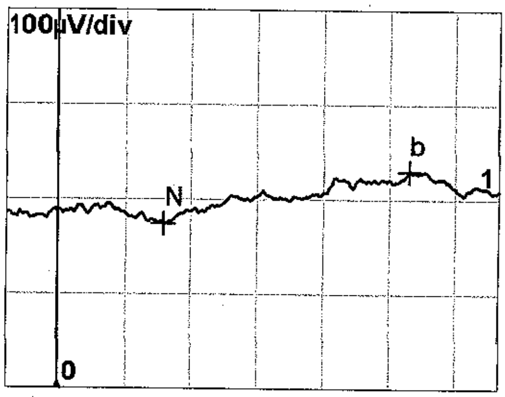

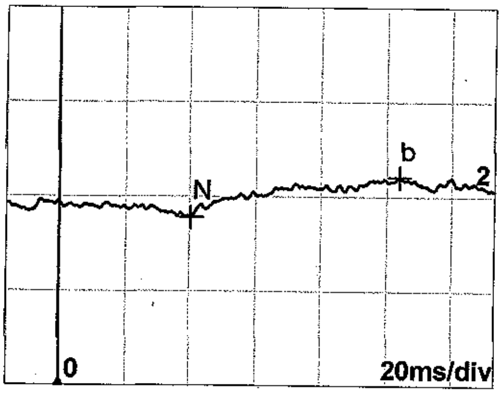

Channel	b [ms]	b-wave
1 R-1	107	56µV
2 L-2	105	42.7µV

Scotopic 3.0 ERG

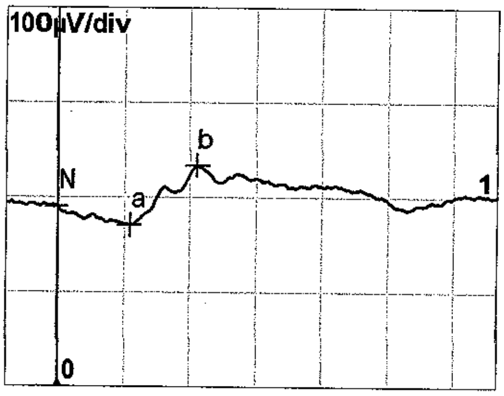

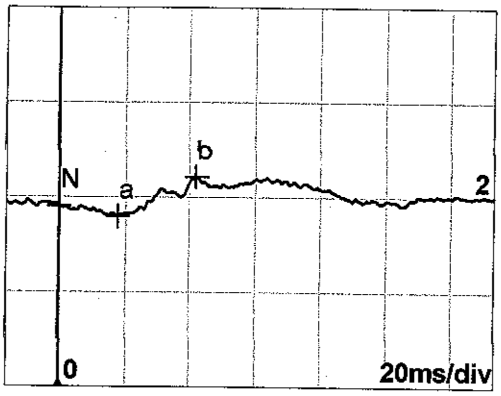

Channel	a [ms]	b [ms]	a-wave	b-wave	b/a
1 R-1	22	43	19.2µV	62.7µV	3.3V
2 L-2	18	43	10.3µV	41.1µV	4V

Scotopic 3.0 oscillatory potentials

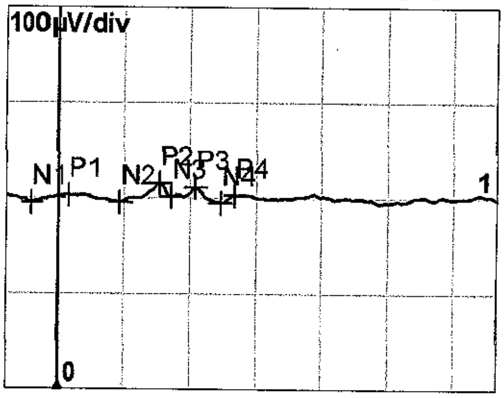

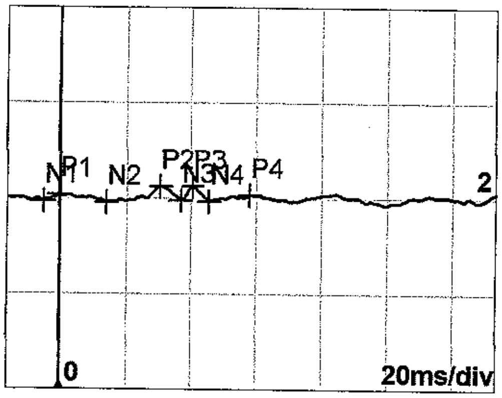

Channe	N1 [ms]	P1 [ms]	N2 [ms]	P2 [ms]	N3 [ms]	P3 [ms]	N4 [ms]	P4 [ms]	OS1	OS2	OS3	OS4	Total
1 R-1	-8	4	19	31	35	42	50	54	7.86µV	20.4µV	9.64µV	8.09µV	0.000V
2 L-2	-5	0	14	31	37	41	46	58	6.49µV	17µV	14.1µV	6.48µV	0.000V

RETIport32

HEBEI EYE HOSPITAL
(No.399 Quanbeidong Rd.xingtai)

Patient: ZC-CEHN-7, CHen, 7/11/2023
Tested: 7/20/2023 9:25:25 PM
ID:
Sex/Age: M/0
Operator:
Electrode: NEEDLE Thread
Pupil Size: 2 dil.

Photopic 3.0 ERG

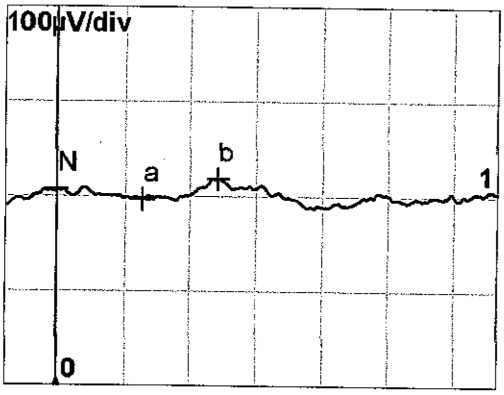

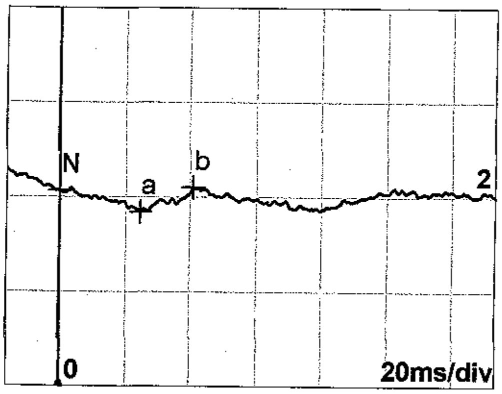

Channel	a [ms]	b [ms]	a-wave	b-wave
1 R-1	26	49	10.3µV	21.8µV
2 L-2	25	41	22.5µV	25.8µV

Photopic 3.0 flicker

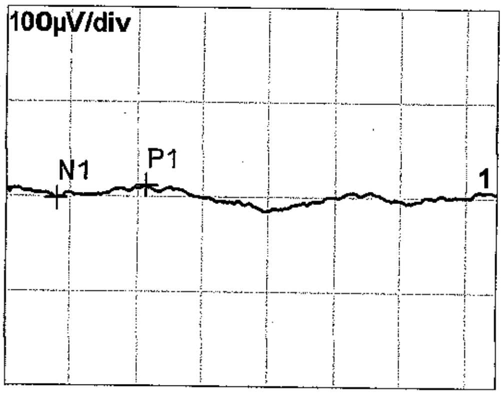

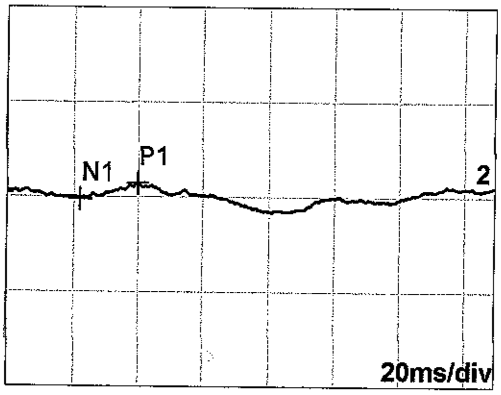

Channel	N1 [ms]	P1 [ms]	V1	N1-P1
1 R-1	16	43	9.96µV	13.5µV
2 L-2	22	40	8.81µV	16.9µV

RETIport32	HEBEI EYE HOSPITAL<br>(No.399 Quanbeidong Rd.xingtai)		
	Patient: ZC-CEHN-8, CHen, 7/11/2023<br>Tested: 7/20/2023 11:03:29 PM<br>ID:	Sex/Age: M/0<br>Operator:	Electrode: NEEDLE Thread<br>Pupil Size: 2 dil.

Scotopic 0.01 ERG

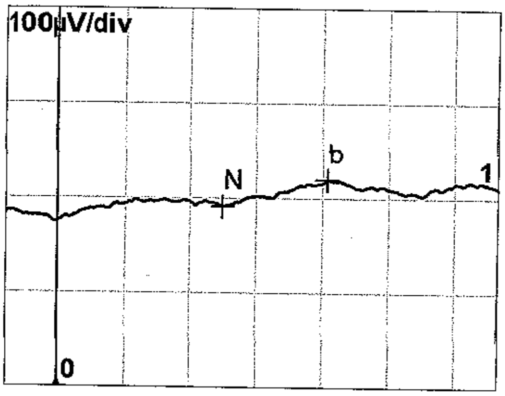

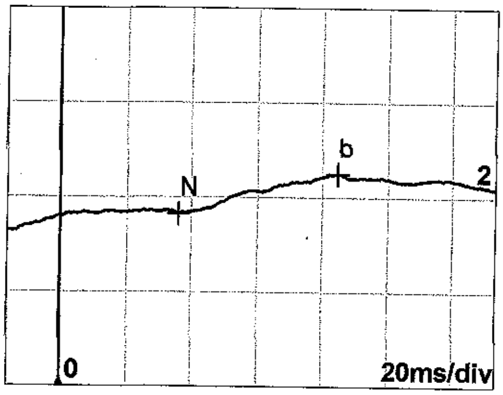

Channel	b [ms]	b-wave
1 R-1	82	28.4µV
2 L-2	85	42.1µV

Scotopic 3.0 ERG

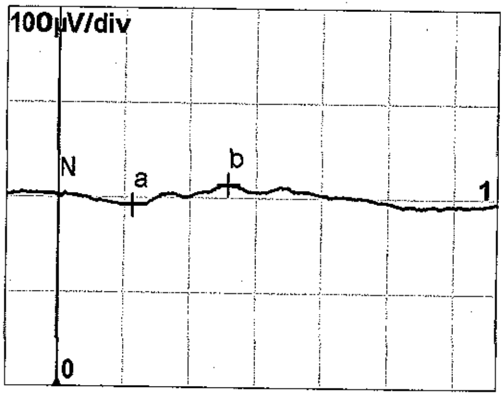

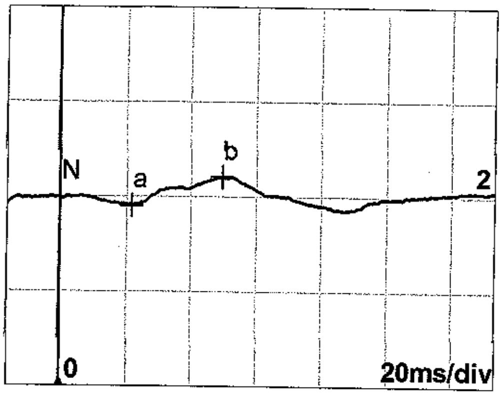

Channel	a [ms]	b [ms]	a-wave	b-wave	b/a
1 R-1	23	52	10.5µV	21.6µV	2V
2 L-2	22	50	8.38µV	30.2µV	3.6V

Scotopic 3.0 oscillatory potentials

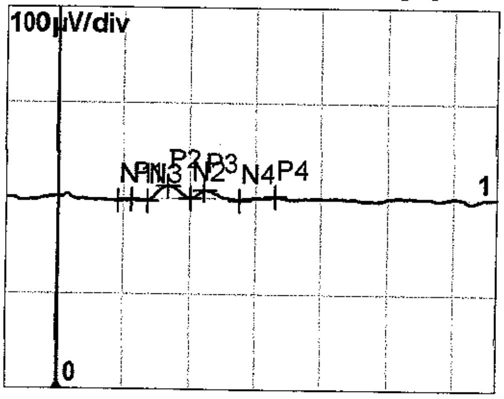

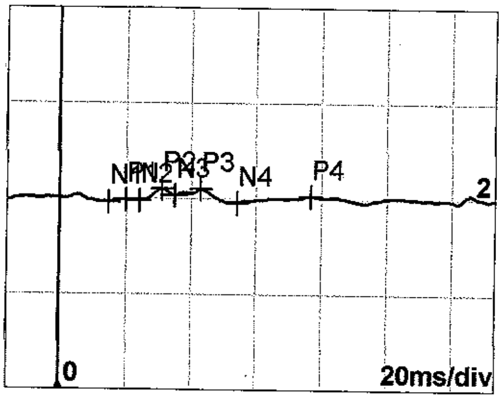

Channel	N1 [ms]	P1 [ms]	N2 [ms]	P2 [ms]	N3 [ms]	P3 [ms]	N4 [ms]	P4 [ms]	OS1	OS2	OS3	OS4	Total
1 R-1	19	23	41	34	27	45	55	66	1.33µV	12.8µV	11.2µV	4.44µV	0.000V
2 L-2	15	20	24	32	36	43	55	77	2.28µV	12.1µV	6.59µV	7.31µV	0.000V

RETIport32

HEBEI EYE HOSPITAL
(No.399 Quanbeidong Rd.xingtai)

Patient: ZC-CEHN-8, CHen, 7/11/2023
Tested: 7/20/2023 11:03:29 PM
ID:
Sex/Age: M/0
Operator:
Electrode: NEEDLE Thread
Pupil Size: 2 dil.

Photopic 3.0 ERG

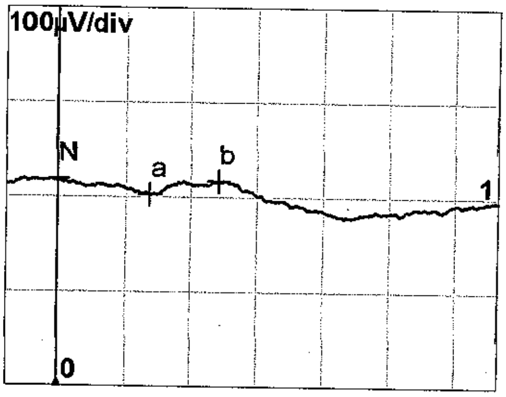

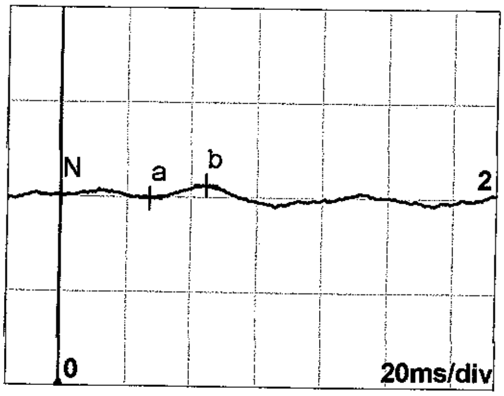

Channel	a [ms]	b [ms]	a-wave	b-wave
1 R-1	28	49	15.6µV	14.4µV
2 L-2	28	45	3.3µV	14µV

Photopic 3.0 flicker

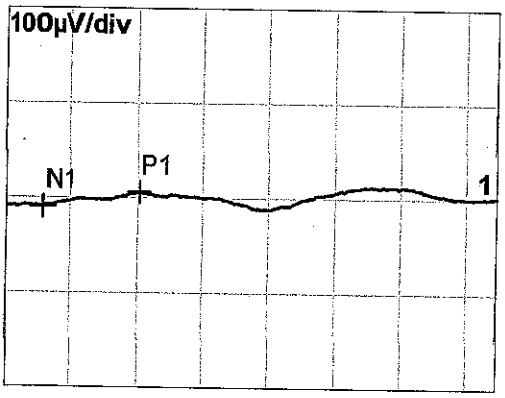

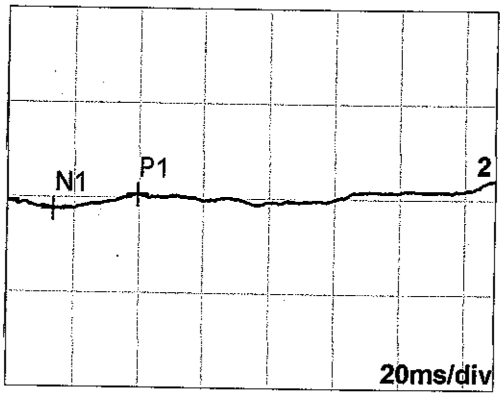

Channel	N1 [ms]	P1 [ms]	V1	N1-P1
1 R-1	12	41	1.06µV	15.5µV
2 L-2	14	40	8.75µV	15.7µV

RETIport32

HEBEI EYE HOSPITAL
(No.399 Quanbeidong Rd.xingtai)

Patient:	ZC-CEHN-13, CHen, 7/11/2023		Electrode: NEEDLE Thread
Tested:	7/20/2023 8:17:43 PM	Sex/Age: M/0	Pupil Size: 2 dil.
ID:		Operator:	

Scotopic 0.01 ERG

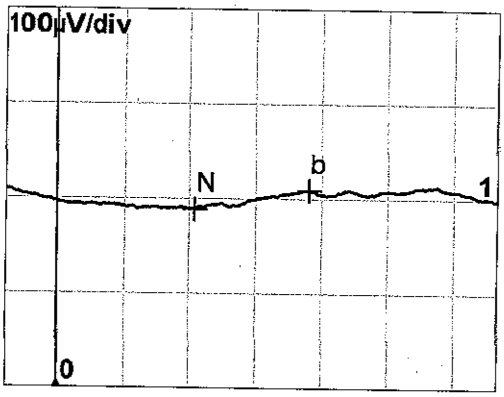

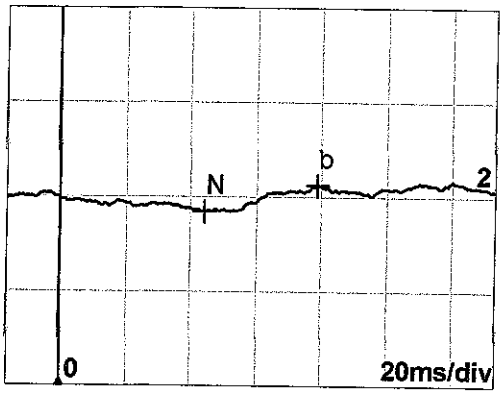

Channel	b [ms]	b-wave
1 R-1	77	20.1μV
2 L-2	79	28.3μV

Scotopic 3.0 ERG

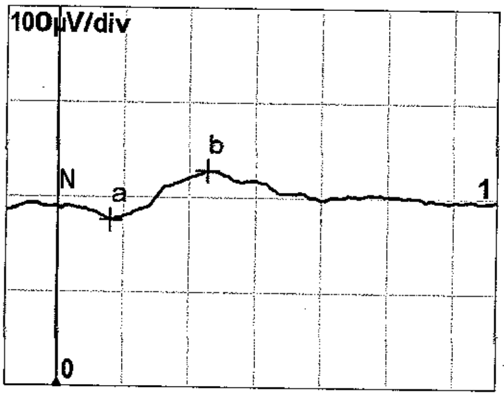

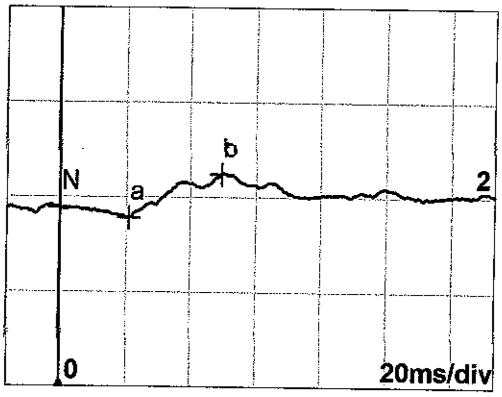

Channel	a [ms]	b [ms]	a-wave	b-wave	b/a
1 R-1	16	46	13.8μV	52.5μV	3.8V
2 L-2	21	50	10.8μV	46.1μV	4.3V

Scotopic 3.0 oscillatory potentials

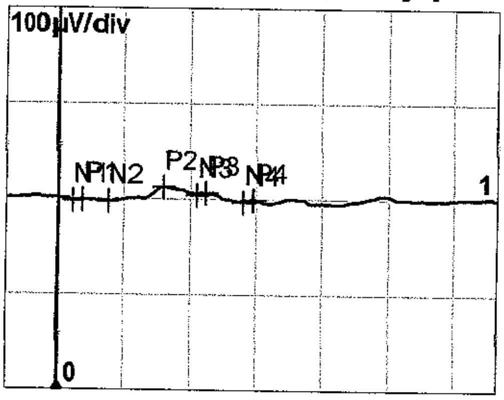

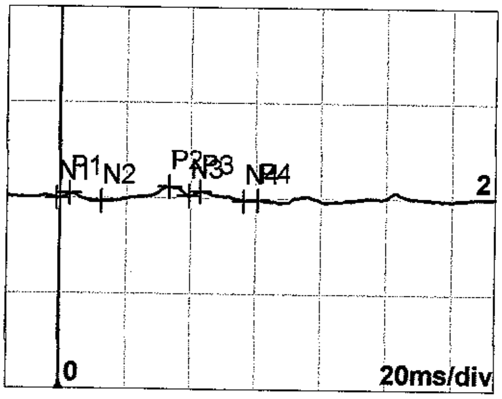

Channel	N1 [ms]	P1 [ms]	N2 [ms]	P2 [ms]	N3 [ms]	P3 [ms]	N4 [ms]	P4 [ms]	OS1	OS2	OS3	OS4	Total
1 R-1	5	8	15	32	43	45	57	60	1.1μV	15.1μV	1.86μV	2.01μV	0.000V
2 L-2	-1	3	13	34	40	43	57	61	4.97μV	15.6μV	4.74μV	964nV	0.000V

RETIport32	HEBEI EYE HOSPITAL<br>(No.399 Quanbeidong Rd.xingtai)		
	Patient: ZC-CEHN-13, CHen, 7/11/2023<br>Tested: 7/20/2023 8:17:43 PM<br>ID:	Sex/Age: M/0<br>Operator:	Electrode: NEEDLE Thread<br>Pupil Size: 2 dil.

Photopic 3.0 ERG

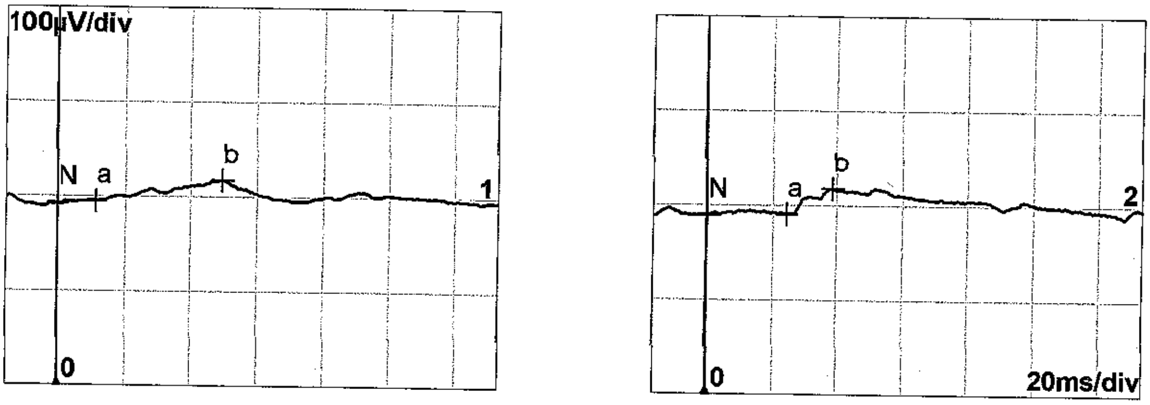

Channel	a [ms]	b [ms]	a-wave	b-wave
1 R-1	11	50	2.31µV	22.6µV
2 L-2	25	39	916nV	27.3µV

Photopic 3.0 flicker

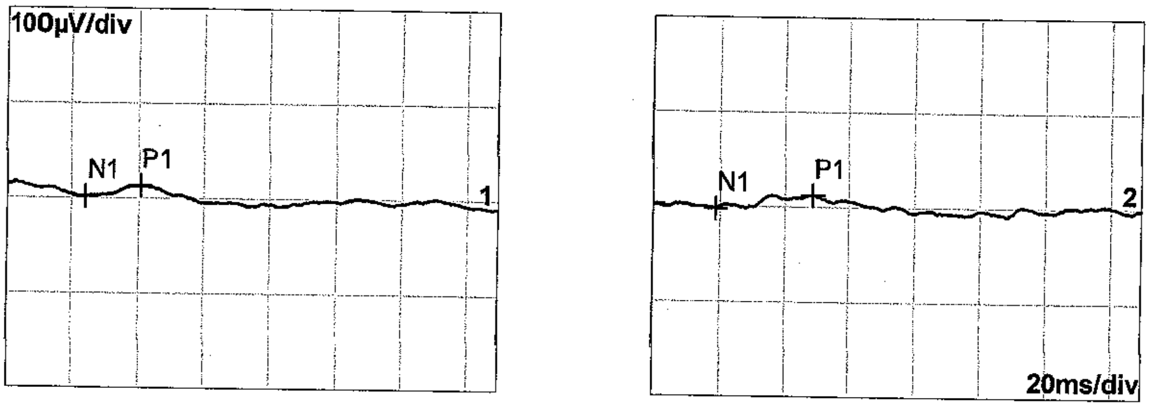

Channel	N1 [ms]	P1 [ms]	V1	N1-P1
1 R-1	24	41	13.8µV	11.9µV
2 L-2	19	49	4.79µV	14.5µV

RETIport32	HEBEI EYE HOSPITAL<br>(No.399 Quanbeidong Rd.xingtai)

Patient: ZC-CEHN-2, CHen, 7//2/2023
Tested: 7/12/2023 7:53:17 PM
ID:
Sex/Age: M/0
Operator:
Electrode: NEEDLE Thread
Pupil Size: 2 dil.

Scotopic 0.01 ERG

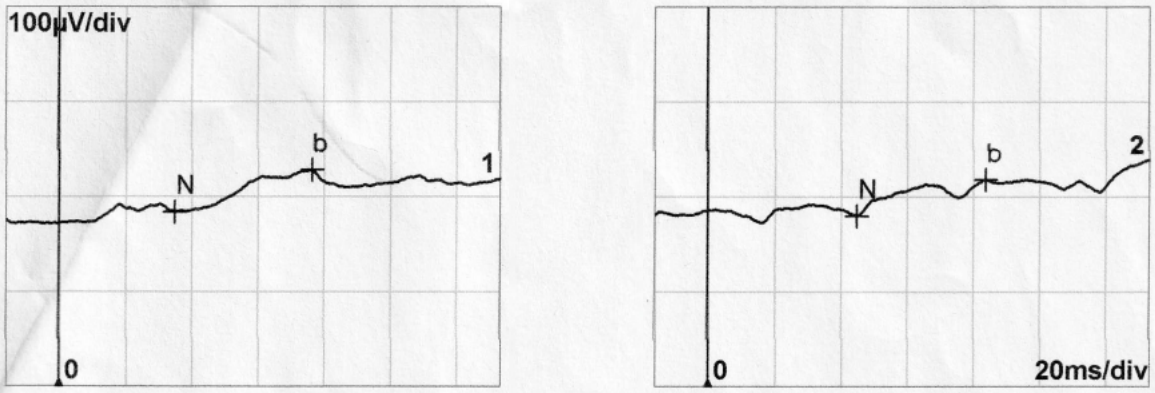

Channel	b [ms]	b-wave
1 R-1	77	43.9µV
2 L-2	84	38.2µV

Scotopic 3.0 ERG

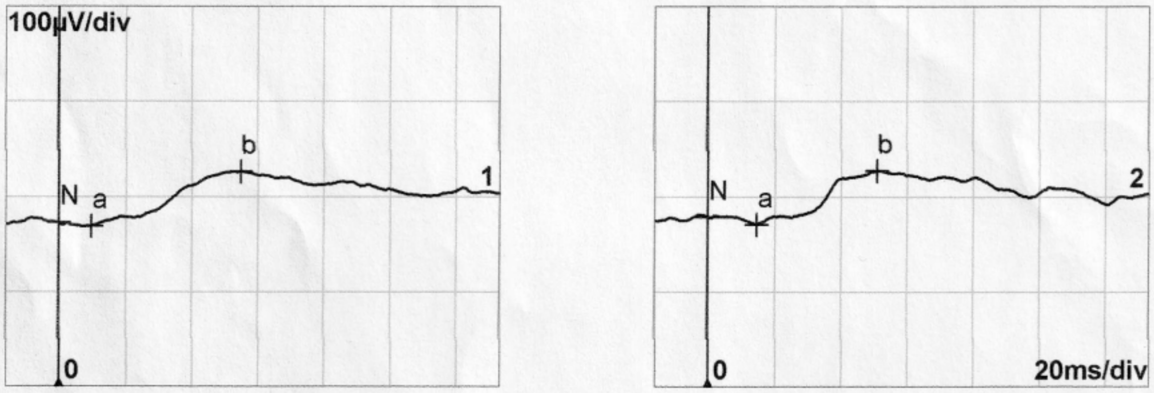

Channel	a [ms]	b [ms]	a-wave	b-wave	b/a
1 R-1	10	55	3.7µV	56.9µV	15V
2 L-2	15	51	7.58µV	56.2µV	7.4V

Scotopic 3.0 oscillatory potentials

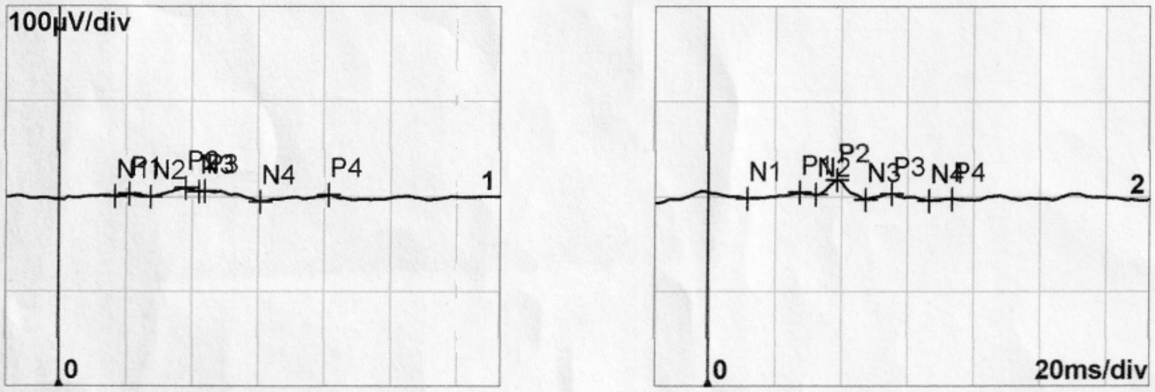

Channe	N1 [ms]	P1 [ms]	N2 [ms]	P2 [ms]	N3 [ms]	P3 [ms]	N4 [ms]	P4 [ms]	OS1	OS2	OS3	OS4	Total
1 R-1	17	21	28	38	43	44	61	82	2.85µV	9.18µV	586nV	7.3µV	0.000V
2 L-2	12	28	33	39	48	56	67	74	6.65µV	15.4µV	6.23µV	1.92µV	0.000V

RETIport32	HEBEI EYE HOSPITAL<br>(No.399 Quanbeidong Rd.xingtai)

Patient: ZC-CEHN-5, CHen, 7/1'2/2023
Tested: 7/12/2023 8:53:00 PM
ID:

Sex/Age: M/0
Operator:

Electrode: NEEDLE Thread
Pupil Size: 2 dil.

Scotopic 0.01 ERG

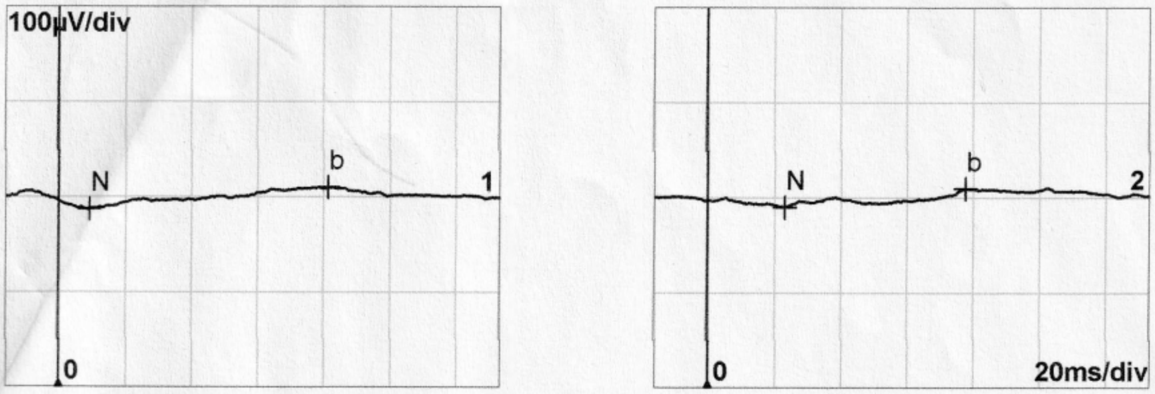

Channel	b [ms]	b-wave
1 R-1	82	22.9µV
2 L-2	78	19.5µV

Scotopic 3.0 ERG

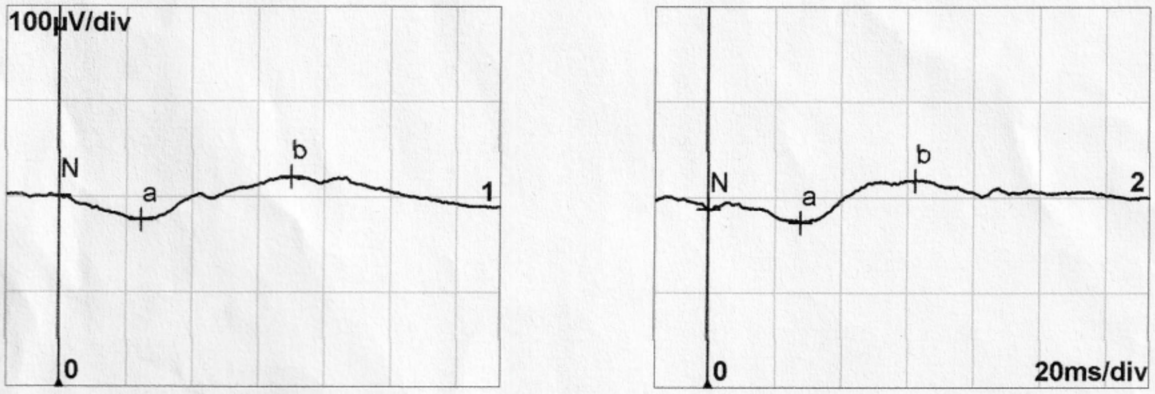

Channel	a [ms]	b [ms]	a-wave	b-wave	b/a
1 R-1	25	70	25.4µV	45.2µV	1.8V
2 L-2	28	63	13.6µV	44µV	3.2V

Scotopic 3.0 oscillatory potentials

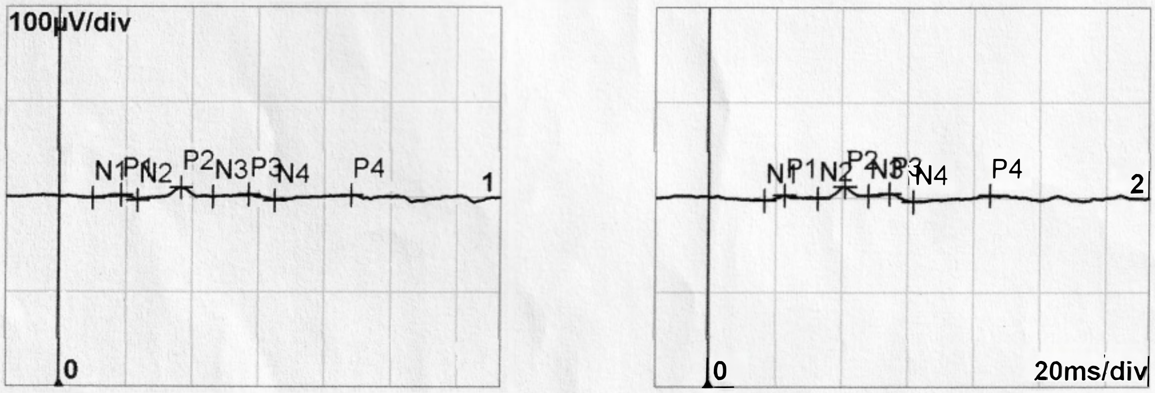

Channel	N1 [ms]	P1 [ms]	N2 [ms]	P2 [ms]	N3 [ms]	P3 [ms]	N4 [ms]	P4 [ms]	OS1	OS2	OS3	OS4	Total
1 R-1	10	19	24	37	47	58	65	89	3.1µV	13.3µV	2.49µV	5.69µV	0.000V
2 L-2	17	23	33	41	48	55	62	85	5.79µV	12.1µV	2.08µV	7.06µV	0.000V

RETIport32

ROLAND CONSULT

HEBEI EYE HOSPITAL
(No.399 Quanbeidong Rd.xingtai)

Patient: ZC. CHEN 9, 7/12/2023
Tested: 7/12/2023 7:12:07 PM
ID:

Sex/Age: M/0
Operator:

Electrode: NEEDLE Thread
Pupil Size: 2 dil

Scotopic 0.01 ERG

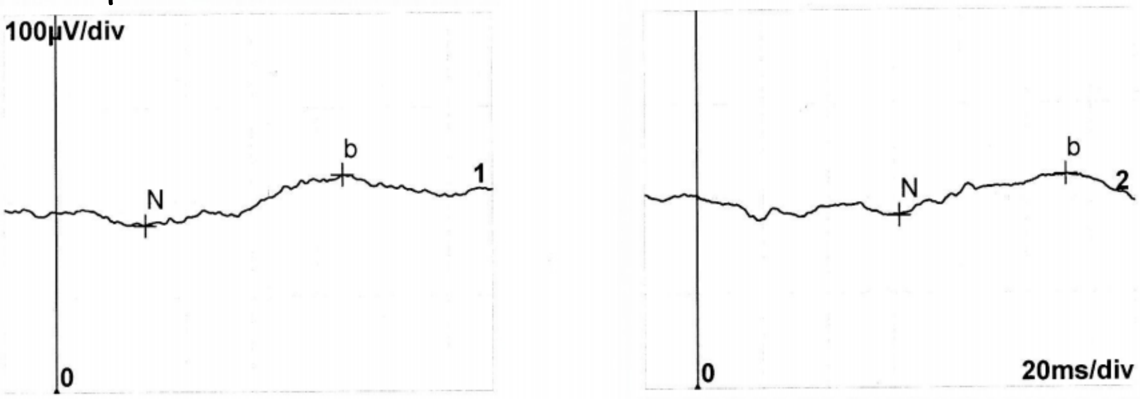

Channel	b [ms]	b-wave
1 R-1	88	53.1µV
2 L-2	113	42.3µV

Scotopic 3.0 ERG

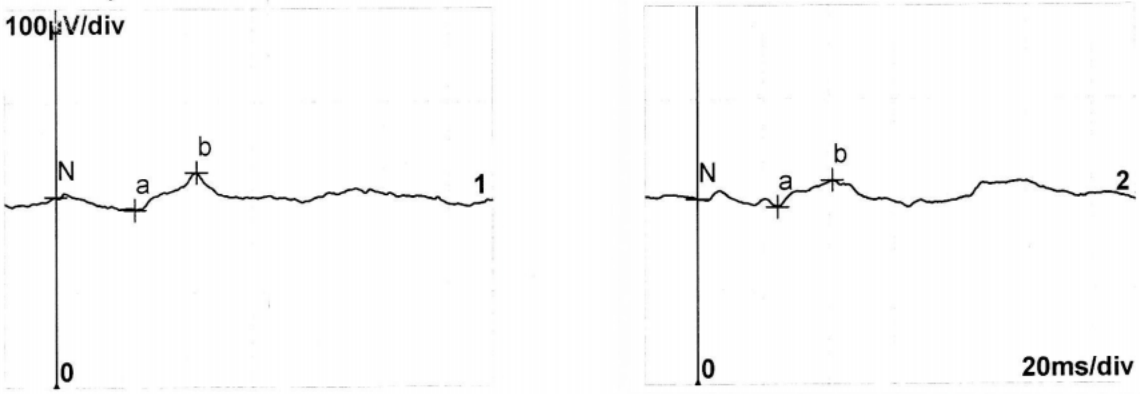

Channel	a [ms]	b [ms]	a-wave	b-wave	b/a
1 R-1	24	43	14.3µV	39.7µV	2.8V
2 L-2	25	41	8.57µV	28.3µV	3.3V

Scotopic 3.0 oscillatory potentials

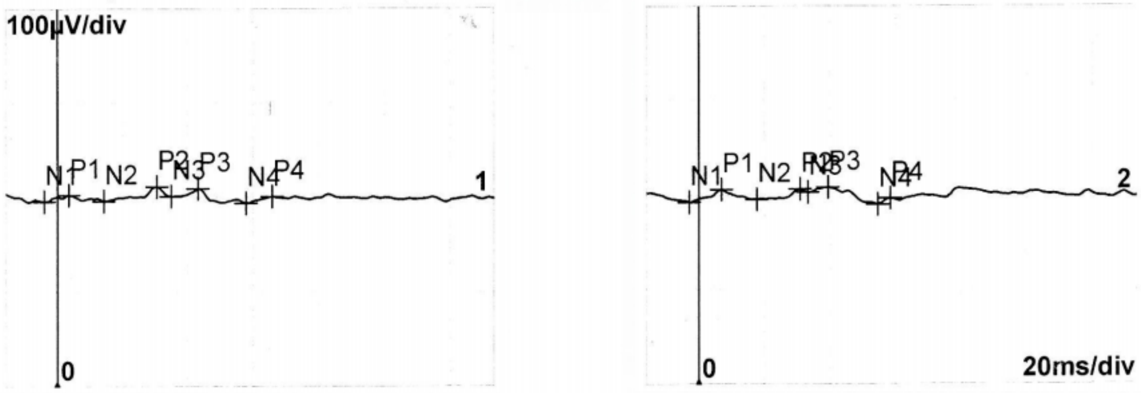

Channe	N1 [ms]	P1 [ms]	N2 [ms]	P2 [ms]	N3 [ms]	P3 [ms]	N4 [ms]	P4 [ms]	OS1	OS2	OS3	OS4	Total
1 R-1	-4	4	14	31	35	43	58	66	6.95µV	15.1µV	8.22µV	7.01µV	0.000V
2 L-2	-3	7	18	31	33	40	55	59	13.3µV	10.6µV	5.11µV	6.15µV	0.000V

RETIport32	HEBEI EYE HOSPITAL<br>(No.399 Quanbeidong Rd.xingtai)				
	Patient:	ZC-CEHN-2, CHen, 7/20/2023			Electrode: NEEDLE Thread
	Tested:	7/20/2023 8:18:07 PM	Sex/Age:	M/0	Pupil Size: 2 dil.
	ID:		Operator:		

Scotopic 0.01 ERG

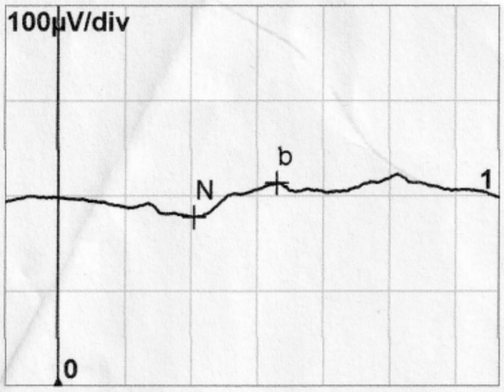

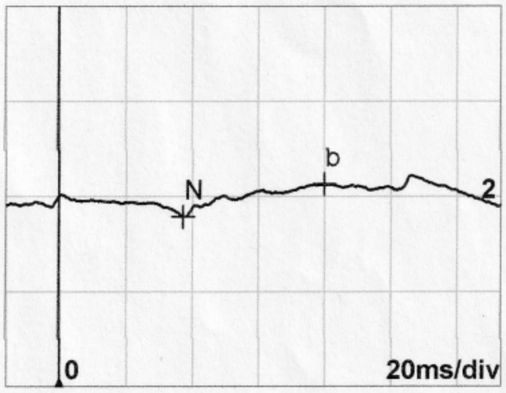

Channel	b [ms]	b-wave
1 R-1	66	36.2µV
2 L-2	80	34µV

Scotopic 3.0 ERG

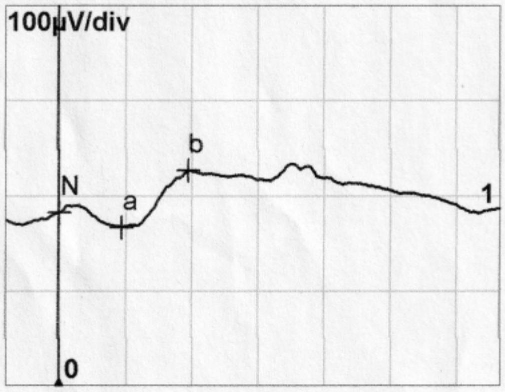

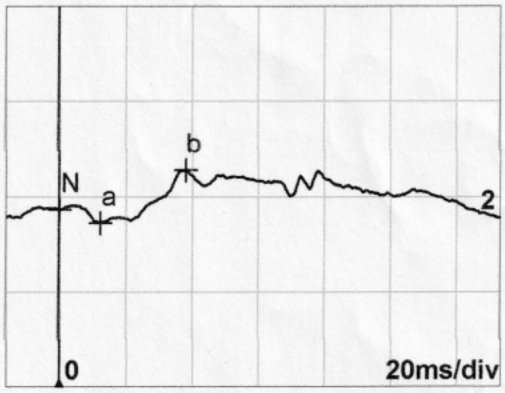

Channel	a [ms]	b [ms]	a-wave	b-wave	b/a
1 R-1	19	39	16.1µV	60µV	3.7V
2 L-2	13	38	14.1µV	55.7µV	4V

Scotopic 3.0 oscillatory potentials

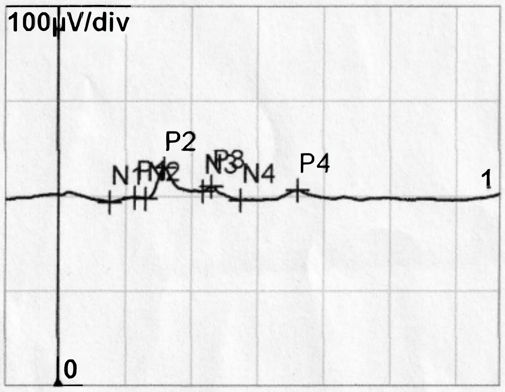

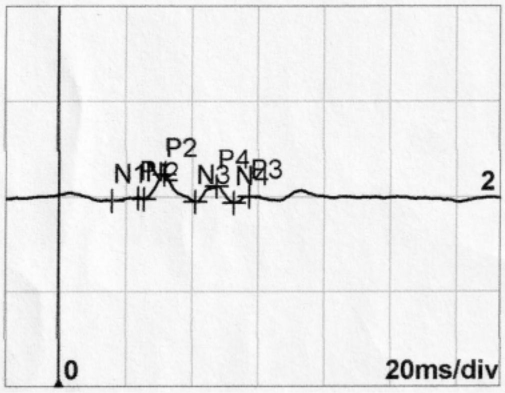

Channe	N1 [ms]	P1 [ms]	N2 [ms]	P2 [ms]	N3 [ms]	P3 [ms]	N4 [ms]	P4 [ms]	OS1	OS2	OS3	OS4	Total
1 R-1	16	23	26	32	44	46	55	73	5.22µV	32.9µV	4.93µV	10.6µV	0.000V
2 L-2	16	24	26	32	41	58	53	48	3.1µV	25.3µV	5.66µV	17.4µV	0.000V

RETIport32

ROLAND CONSULT

HEBEI EYE HOSPITAL
(No.399 Quanbeidong Rd.xingtai)

Patient: ZC-CEHN-5, CHen, 7/20/2023
Tested: 7/20/2023 7:05:52 PM
ID:

Sex/Age: M/0
Operator:

Electrode: NEEDLE Thread
Pupil Size: 2 dil.

Scotopic 0.01 ERG

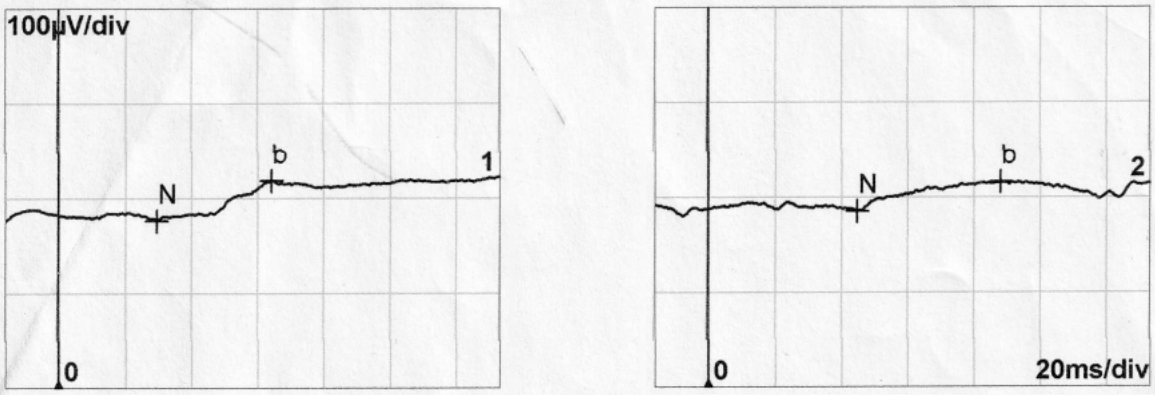

Channel	b [ms]	b-wave
1 R-1	65	42µV
2 L-2	89	30.8µV

Scotopic 3.0 ERG

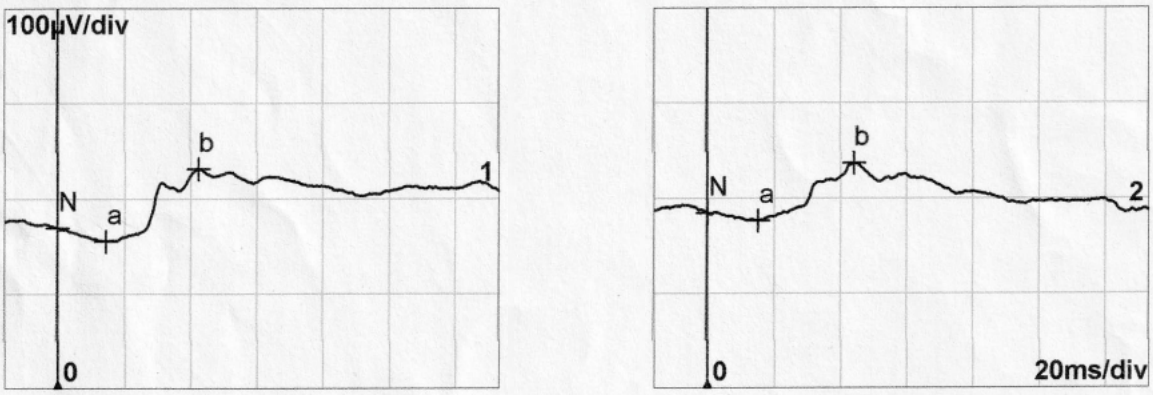

Channel	a [ms]	b [ms]	a-wave	b-wave	b/a
1 R-1	15	43	14.1µV	76.3µV	5.4V
2 L-2	16	45	7.67µV	60.6µV	7.9V

Scotopic 3.0 oscillatory potentials

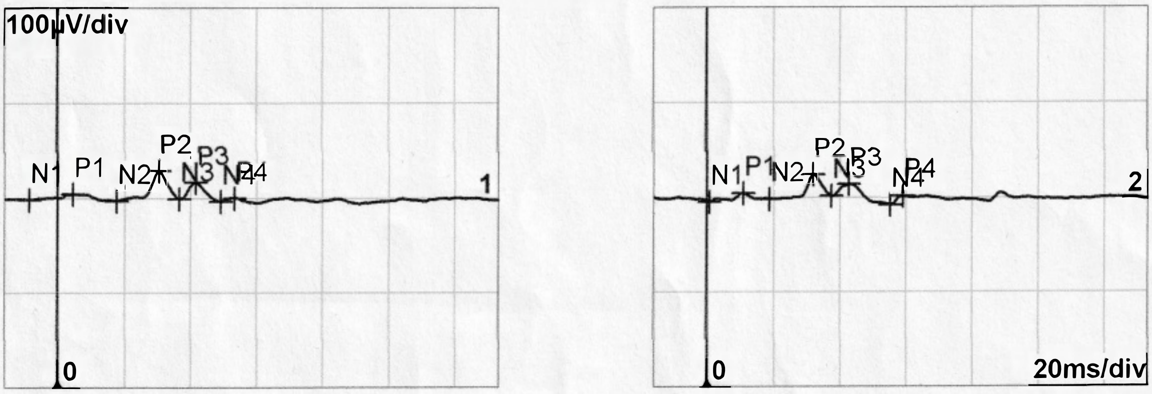

Channel	N1 [ms]	P1 [ms]	N2 [ms]	P2 [ms]	N3 [ms]	P3 [ms]	N4 [ms]	P4 [ms]	OS1	OS2	OS3	OS4	Total
1 R-1	-9	5	18	31	37	42	50	54	6.08µV	31.7µV	17.3µV	3.42µV	0.000V
2 L-2	1	11	19	32	38	43	56	60	8.89µV	25.9µV	12µV	9.64µV	0.000V

RETIport32	HEBEI EYE HOSPITAL<br>(No.399 Quanbeidong Rd.xingtai)
	Patient: ZC-CEHN-9, CHen, 7/20/2023<br>Tested: 7/20/2023 7:34:08 PM Sex/Age: M/0<br>ID: Operator:<br>Electrode: NEEDLE Thread<br>Pupil Size: 2 dil.

Scotopic 0.01 ERG

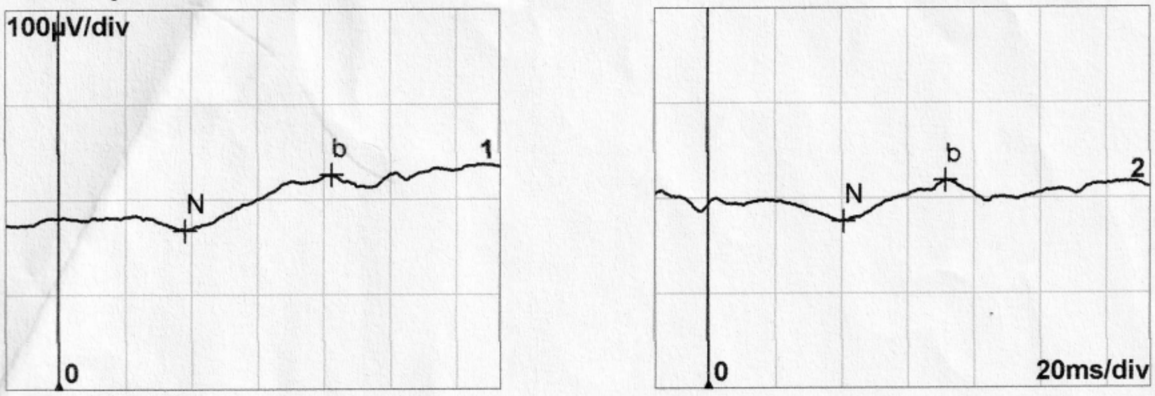

Channel	b [ms]	b-wave
1 R-1	83	58.3µV
2 L-2	72	42.8µV

Scotopic 3.0 ERG

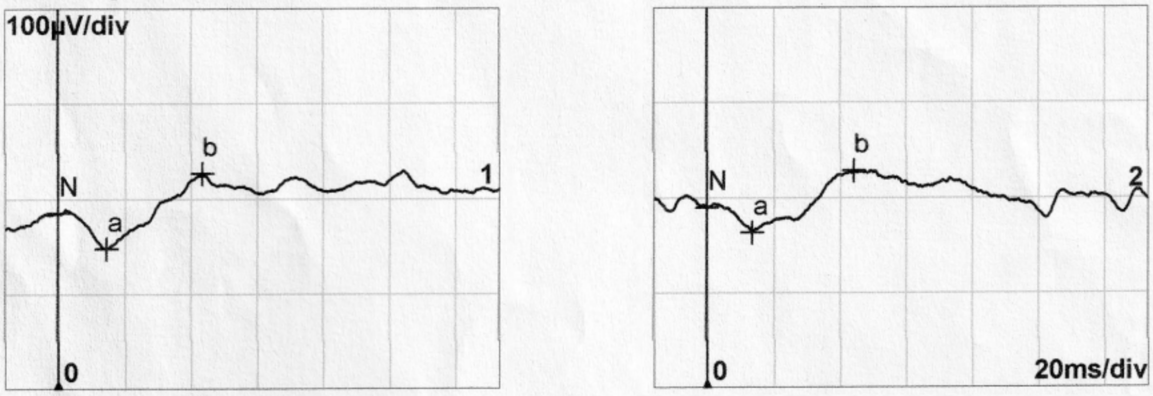

Channel	a [ms]	b [ms]	a-wave	b-wave	b/a
1 R-1	15	44	37.7µV	79.1µV	2.1V
2 L-2	14	45	26.5µV	65µV	2.5V

Scotopic 3.0 oscillatory potentials

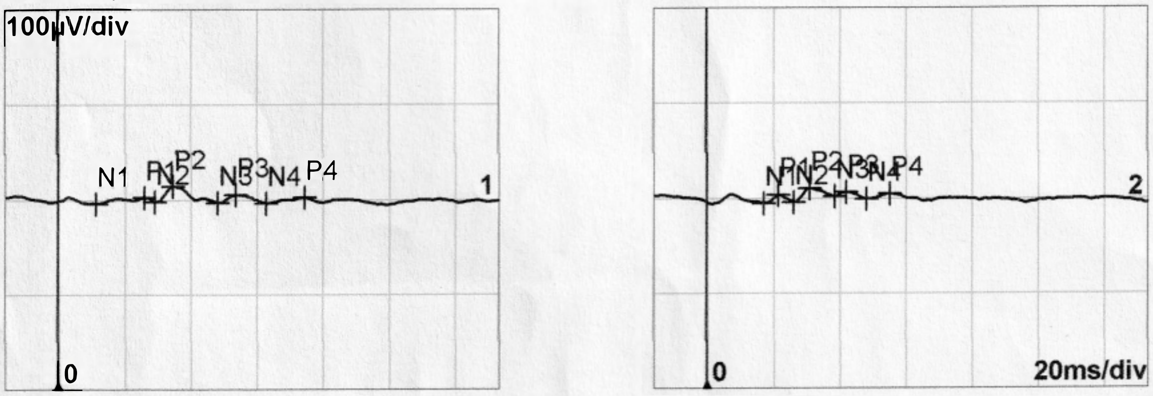

Channe	N1 [ms]	P1 [ms]	N2 [ms]	P2 [ms]	N3 [ms]	P3 [ms]	N4 [ms]	P4 [ms]	OS1	OS2	OS3	OS4	Total
1 R-1	11	26	29	35	48	54	63	75	6.93µV	16.2µV	8.67µV	6.57µV	0.000V
2 L-2	17	22	26	31	39	42	48	55	8.86µV	14.8µV	4.2µV	5.59µV	0.000V
